# Chinese herbal medicine xuebijing injection for acute pancreatitis: An overview of systematic reviews

**DOI:** 10.3389/fphar.2022.883729

**Published:** 2022-08-10

**Authors:** Fengya Zhu, Shao Yin, Li Zhou, Zimeng Li, Hui Yan, Yue Zhong, Xiaohan Wu, Biao Luo, Lanying Yang, Daohui Gan, Lvyu Deng, Deya Che, Liuying Li

**Affiliations:** ^1^ Traditional Chinese Medicine Department, Zigong First People’s Hospital, Zigong, China; ^2^ Clinical Medical School, Hospital of Chengdu University of Traditional Chinese Medicine, Chengdu, China; ^3^ Acupuncture and Tuina School, The Third Teaching Hospital, Chengdu University of Traditional Chinese Medicine, Chengdu, China

**Keywords:** Chinese herbal medicine, Xuebijing injection, acute pancreatitis, overview, systematic reviews

## Abstract

**Background:** At present, a number of systematic reviews (SRs) on Xuebijing injection (a patent in China) in the treatment of acute pancreatitis (AP) or severe acute pancreatitis (SAP) have been published. However, the quality of evidence is uneven and has not been comprehensively evaluated.

**Aim:** We evaluated the efficacy of Xuebijing injection for AP/SAP through an overview of SR, and to provide a scientific basis for its effectiveness and safety.

**Methods:** We searched Cochrane Library, Embase, PubMed, SinoMed, CNKI, Wanfang, and VIP comprehensively. The retrieval period from inception to 30 November 2021, and the two reviewers independently complete the literature retrieval, data extraction and evaluation. The Assessing the Methodological Quality of Systematic Reviews 2 (AMSTAR-2) and the Preferred Reporting Item for Systematic Review and Meta-analysis (PRISMA) were used to evaluate the methodological quality and reporting quality of the SRs, respectively. The Grading of Recommendations Assessment, Development, and Evaluation (GRADE) tool was used to evaluate the quality grading of outcomes and the risk of bias in SRs was evaluated by ROBIS Tool. Finally, the RCTs involved in SRs were synthesized. Stata15.1 was used for quantitative analysis of total effectiveness rate, time until relief of abdominal pain, time until relief of abdominal distension, and serum amylase level.

**Results:** Nine eligible SRs were included, including 92 RCTs and 6,837 participants. The quality of SRs was relatively good, and the manuscript structures were relatively complete. However, the methodological quality of SRs was low or critically low. RoB rated 5 SRs as low risk of bias and 4 SRs as high risk of bias. In GRADE, a total of 47 results were included in the 9 SRs, of which 5 results (10.64%) were moderate quality, 22 results (46.81%) were low quality, and 20 results (42.55%) were very low quality. The results of data synthesis showed that Xuebijing injection combined treatment increased the total effectiveness rate of AP patients (RR = 1.19, 95% CI 1.17–1.23, *p* < 0.0001), and there was no heterogeneity between studies (I^2^ = 0.0%, *p* = 0.589). Compared with the control group, Xuebijing injection group shortened the abdominal pain and distension relief time in AP patients (WMD = −1.69, 95% CI −1.88–−1.50, *p* < 0.0001; WMD = −1.48, 95% CI −1.74–−1.23, *p* < 0.0001), with high heterogeneity (I^2^ = 84.3%, *p* = 0.000; I^2^ = 72.2%, *p* = 0.000). Serum amylase level was also reduced (WMD = −2.06, 95% CI −2.47–−1.64, *p* < 0.0001), with significant heterogeneity (I^2^ = 71.6%, *p* = 0.000). A total of one SR reported adverse drug reaction (ADR), no ADRs were observed in the control group.

**Conclusion:** Although the quality of the evidence is not high, it can still reflect the clinical value of Xuebijing injection as an analgesic and anti-inflammatory traditional Chinese medicine in the treatment of AP/SAP. Therefore, future clinical studies should focus on the long-term efficacy and adverse reactions of drugs.

**Systematic Review Registration:** (website), identifier (registration number).

## 1 Introduction

Globally, the incidence of acute pancreatitis (AP) is increasing, and it is one of the most common gastrointestinal disorders in emergency hospital admissions (van Dijk et al., 2017). Eighty percent of the patients had mild disease, which could be spontaneously remitted without serious complications, but 20% of the patients had severe acute pancreatitis (SAP), which may lead to serious complications and high mortality (Lund et al., 2006). Mortality rates are similar between several etiologies of AP, including gallstone-related and alcohol-induced AP (Andersen et al., 2008), hypertriglyceridemia and alcohol-induced AP (Goyal et al., 2016). Multiple organ dysfunction (MODS) and even persistent multiple organ failure (PMOF) are the leading causes of AP mortality. The etiology and pathogenesis of AP have been thoroughly studied. The important theories concerning pathogenesis include the bile-pancreatic duct common pathway theory, pancreatic autodigestion theory, gallstone migration theory, enzyme activation theory, kinin and complement systematic activation theory, microcirculation disturbance theory, leukocyte excessive activation theory, and pancreatic acinar cell apoptosis and necrosis theory (Wang et al., 2009), but the theories of pathogenicity are diverse and highly controversial. At present, the clinical treatment of AP mainly includes supportive care, nutrition, prophylactic antibiotics, cholecystectomy, symptomatic management of complications (Greenberg et al., 2016; Garber et al., 2018), and new drug therapies (including anti-secretants, protease inhibitors, anti-inflammatory agents and antioxidants, etc.) (Kambhampati et al., 2014). However, due to the rapid progression of the disease, many drug therapies have not yet shown therapeutic benefits, and there is a great need and prospect for the development of effective drug therapies for AP/SAP.

Xuebijing injection (a patent in China) is a compound injection based on traditional Chinese herbs and developed under the guidance of the theory of “combination of bacteria, toxin and inflammation,” and its main ingredients include Paeonia lactiflora Pal. (Shaoyao), Ligusticum chuanxiong hort (Chuanxiong), Salvia miltiorrhiza Bunge (Danshen), Carthamus tinctorius L. (Honghua), and Angelica sinensis (Danggui). At present, Xuebijing injection has been approved by the China Food and Drug Administration for the treatment of sepsis and MODS (Yin and Li, 2014).

Multiple systematic reviews (SRs) have reported the efficacy and safety of Xuebijing injection to treat AP/SAP, but the quality of evidence is uneven, and low-quality SR may mislead clinical decision-making. Overview is a new way to gather disparate SR data, reassess methodological quality, and synthesize individual data (Pollock et al., 2016). To the best of our knowledge, different SR evidence for the treatment of AP/SAP by Xuebijing injection has not been comprehensively evaluated. Therefore, we will conducte a comprehensive evaluation of SRs of Xuebijing injection for AP/SAP, and to provide a scientific basis for its effectiveness and safety.

## 2 Methods

### 2.1 Search strategy

Relevant articles were searched in the following seven databases from inception to 30 November 2021: Cochrane Library, Embase, PubMed, SinoMed, CNKI, Wan-fang Database, and VIP Database. The key search words included “xuebijing,” “xuebijing injection,” “pancreatitis,” “acute pancreatitis,” “pancreatitis, acute hemorrhagic,” “systematic review,” “systematic evaluation,” and “meta-analysis.” The search strategy was adjusted according to the characteristics of each database. In addition, we manually searched for relevant review article citations. The search strategies are shown in the Supplementary Tables.

### 2.2 Inclusion criteria

All articles met the following inclusion criteria: 1) Study design and participants: SR was based on randomized controlled trials (RCTs). In addition, SR used meta-analysis as a statistical method to analyze and summarize the results of the included studies. Participants with a clear diagnosis of AP/SAP (meeting one of the accepted diagnostic criteria), age, sex, race, nationality, and course of illness were not limited; 2) Study intervention and comparison: the treatment group was mainly treated with Xuebijing injection or combined with other treatments, while the control group used any other treatment methods except Xuebijing injection; 3) Study outcomes included total effectiveness rate, time until relief of abdominal pain, time until relief of abdominal distension, serum amylase level, white blood cell recovery time, hospital stay, IL-6 level, IL-8 level, TNF-α level, and CRP (including at least one outcome).

Total effectiveness rate = (markedly improved patients + improved patients)/total number of patients. Markedly improved atients were defined as significant remission or disappearance of clinical symptoms and signs and significant improvement or normalization of laboratory indicators after treatment; improved patients were defined as improvement of clinical symptoms and signs and laboratory indicators after treatment.

### 2.3 Exclusion criteria

The following articles were excluded: 1) incomplete data and duplicate papers; 2) not SRs, including general reviews, conference articles, case reports, expert consensus, comments, etc.; 3) Xuebijing injection was not the main therapy.

### 2.4 Study selection and data extraction

The two reviewers (LYD and DHG) performed independent literature retrieval. Preliminary screening was conducted by reading titles and abstracts, and the articles were entered into EndNote X9.1 to eliminate duplicate documents. Then, the full text was read further, and any literature that did not meet the criteria was excluded. Any dispute was settled between the two reviewers, and unresolved issues were settled by a third reviewer (XHW).

Two reviewers (BL and LYY) independently extracted data, including author, publication year, language, condition, sample size, diagnostic criteria, intervention, outcomes, quality assessment methods, and main conclusion. All cross-checks were completed. Any dispute was settled between the two reviewers, and unresolved issues were settled by a third reviewer (ZML).

### 2.5 Quality evaluation

Two reviewers (YZ and HY) independently used AMSTAR-2, PRISMA, ROBIS, and GRADE tools to evaluate the methodological quality, reporting quality, risk of bias, and evidence quality of SRs. Differences in results were negotiated by the two reviewers, and unresolved disagreements were settled by a third reviewer (LYL).

The AMSTAR-2 is a practical methodological quality assessment tool for SR of randomized or nonrandomized trials, with a total of 16 items, and Items 2, 4, 7, 9, 11, 13, and 15 are critical items. These items can critically affect the validity of a review and its conclusion. There are four levels of quality for an SR: high, moderate, low, and critically low. (I) High: none or only one nonkey item does not meet the “high level”; (II) more than one noncritical item does not meet the “moderate level”; (III) only one critical item does not meet, with or without noncritical items, the “low level”; (IV) more than one critical item does not meet the requirements, with or without noncritical items that meet to the “critically low level” (Huan et al., 2018; Zhang et al., 2018).

PRISMA is used to evaluate the reporting quality of SR and contains 27 items (Liberati et al., 2009). Each item is answered with “yes,” “no,” and “partial yes.” Percentages represent the items that qualify for “yes.”

The ROBIS is a tool used to evaluate the risk of bias (RoB) in SR and is divided into three phases. The first phase is optional, and the second phase consists of four key areas: “study eligibility criteria,” “identification and selection of studies,” “data collection and study appraisal,” and “synthesis and findings.” The third phase is based on the evaluation of the four areas in the second stage for comprehensive evaluation, and the SRs are evaluated as “low risk,” “high risk,” and “unclear risk.”(Whiting et al., 2016).

GRADE is applied for evidence quality assessment of included outcomes of SRs. Limitations, inconsistency of results, indirectness of evidence, imprecision and reporting bias can lead to the degradation of evidence quality. The quality of evidence can be classified into four levels: high, moderate, low, and very low. No degradation equates to high quality, one degradation to moderate quality, two degradation to low quality, and three or more degradation to very low quality (Atkins et al., 2004).

### 2.6 Data synthesis and analysis

To better observe the efficacy of Xuebijing injection in the treatment of AP, quantitative data of RCTs involved in SRs were summarized. Stata15.1 is used for data analysis, in which dichotomous variables are represented by risk ratios (RRs) and 95% confidence intervals (CIs), and continuous variables are represented by WMDs and 95% CIs. *p* < 0.05 indicates statistical significance. When the heterogeneity was significant (I^2^ > 50%), the random effect model was used. The funnel plot and Egger’s test were used to test publication bias. Sensitivity analysis is used to verify the stability of the results.

## 3 Results

### 3.1 Search results

**FIGURE 1:**
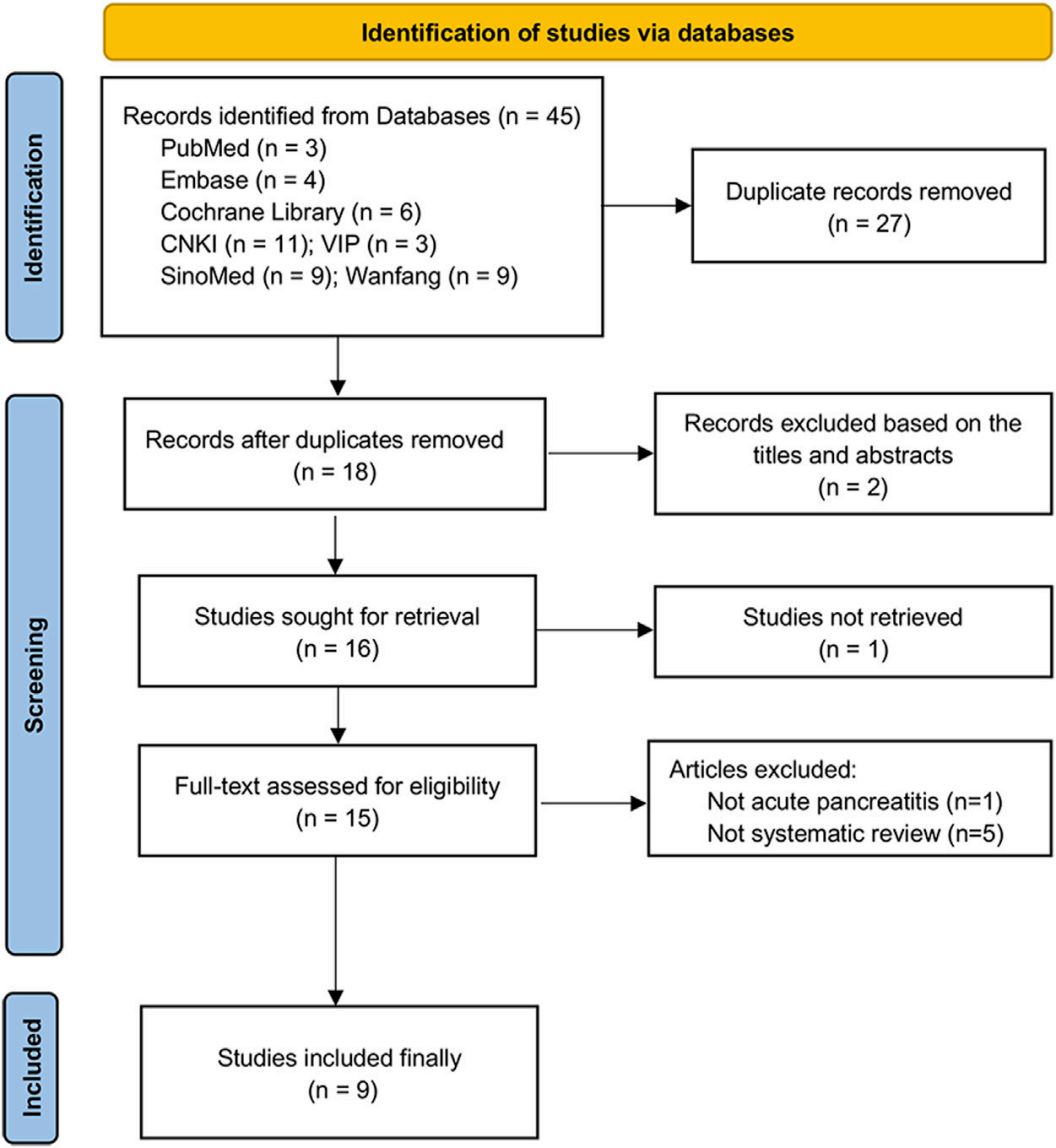
Flow chart of study selection.

We formulated a standardized search strategy based on the characteristics of each database, to accurately assess relevant literature. Forty-five articles were initially screened from seven databases. A total of 27 duplicate references were excluded in Endnote X9.1; furthermore, three articles were excluded after reading the title and abstract, and six articles were excluded by reading the full text. Finally, a total of 9 SRs were included for data analysis (Li et al., 2010; Dong and Huang, 2011; Jie et al., 2014; Zheng et al., 2015; Zhang and Ke, 2016; Ma et al., 2017; Chen et al., 2021a; Xu et al., 2021; Zhou et al., 2021). The literature search and screening process is shown in Figure 1. The list of excluded articles and reasons for exclusions in “full-text assessed for eligibility” are shown in the Supplementary Tables.

### 3.2 Characteristics of included reviews

All SRs were published between 2011 and 2021, of which one was published in English and the remaining eight were published in Chinese. The study included 92 original RCTs involving 6,837 participants after duplicates were removed (Supplementary Material). There are two main diagnostic criteria: Guidelines for Diagnosis and Treatment of Acute Pancreatitis in China (Draft), 2004 and Guidelines for the Diagnosis and Treatment of Acute Pancreatitis (2014 edition)-Chinese Medical Association. Xuebijing injection was used in the treatment group, among which three were combined with ulinastatin (Jie et al., 2014; Ma et al., 2017; Zhou et al., 2021), and six were combined with routine treatment (Li et al., 2010; Dong and Huang, 2011; Zheng et al., 2015; Zhang and Ke, 2016; Chen et al., 2021a; Xu et al., 2021). The control group adopted the same method as the treatment group but without Xuebijing injection. The main results were total effectiveness rate, time until relief of abdominal pain, time until relief of abdominal distension and serum amylase level. Secondary results included white blood cell recovery time, hospital stay, IL-6 level, IL-8 level, TNF-α level, and CRP. Regarding the methodological evaluation tool, five SRs used the Cochrane risk of bias tool (Li et al., 2010; Zhang and Ke, 2016; Chen et al., 2021a; Xu et al., 2021; Zhou et al., 2021), two SRs used the Jadad scale (Jie et al., 2014; Zheng et al., 2015), and two SRs did not mention a specific tool (Dong and Huang, 2011; Ma et al., 2017). Only two SRs mention adverse drug reactions (ADRs); see Table 1 for the characteristics of the included SRs.

**TABLE 1:** Characteristics of the included systematic reviews.

Included studies	Language	Condition	Number of RCTs (participants)	Diagnostic criteria	Intervention (D/F)	Comparison (D/F)	Primary outcomes	Methodological evaluation tool	Adverse drug reaction (number of RCTs, E/C)	Main conclusion
Chen et al. (2021a)	English	AP	23 (1882)	Not reported	Xuebijing injection 100 ml (7-14 days/bid) + Comparison	RT (Not reported)	①②③④⑤⑦⑩	Cochrane risk of bias tool	Not reported	Application of Xuebijing injection on the basis of conventional treatment can improve the outcomes of AP.
Xu et al. (2021)	Chinese	AP	61 (4,868)	2014	Xuebijing injection (7-14 days/Not reported) + Comparison	RT (Not reported)	①②③④⑤⑥⑦⑧⑨⑩	Cochrane risk of bias tool	Not reported	The total effectiveness rate of Xubijing injection combined with routine medicine therapy in the treatment of acute pancreatitis is higher than that of routine medicine therapy alone
Zhou et al. (2021)	Chinese	SAP	10 (633)	2004 + 2014	Xuebijing injection 100 ml (7-14 days/bid) + Comparison	Ulinastatin + RT (Not reported)	①②③④⑤⑦⑨	Cochrane risk of bias tool	Not reported	Combined with Xuebijing injection to treat SAP patients could effectively reduce TNF-α and IL - 6 levels, relieve clinical symptoms and signs, and improve overall clinical efficacy
Ma et al. (2017)	Chinese	AP	11 (893)	2014	Xuebijing injection+ Comparison (Not reported)	Ulinastatin + RT (Not reported)	①②④⑤⑥⑦⑧⑨	Not mention	Not reported	Xuebijing injection combined with Ulinastatin and RT was more effective in the treatment of AP than the control group, but the evidence was insufficient
Zhang and Ke. (2016)	Chinese	SAP	10 (811)	2004	Xuebijing injection 50–100 ml (7-10 days/bid) + Comparison	Raceanisodamine Hydrochloride Injection 20 mg (7 days/bid) Alanyl glutamine injection 20 g (7 days/bid)	⑦⑧⑨	Cochrane risk of bias tool	Not reported	On the basis of RT, Xuebijing injection could significantly improve the levels of various inflammatory factors in SAP.
Zheng et al. (2015)	Chinese	SAP	15 (814)	2004	Xuebijing injection50-100 ml (7-14 days/bid) + Comparison	RT (Not reported)	①⑥	Jadad	Headache, nausea, and a slight rash (1/0)	Xuebijing injection combined with RT for SAP patients had obvious advantages, but there was insufficient evidence
Jie R. et al. (2014)	Chinese	SAP	5 (421)	2004	Xuebijing injection50-100 ml (7 days/Not reported)+ Comparison	Ulinastatin + RT (Not reported)	①②④⑤	Jadad	Not reported	Xuebijing injection could significantly improve the clinical symptoms and objective indications of patients with SAP.
Dond and Huang. (2011)	Chinese	AP	8 (567)	2004	Xuebijing injection + Comparison (Not reported)	RT (Not reported)	②④⑥	Not mention	Not reported	The treatment group could significantly improve the clinical symptoms and shorten the length of hospital stay
Li et al. (2010)	Chinese	SAP	4 (242)	2004	Xuebijing injection + Comparison (Not reported)	RT (Not reported)	②③④⑤	Cochrane risk of bias tool	Not reported	The treatment group could significantly reduce the clinical symptoms and objective indications of patients with SAP.

AuthorAnonymous et al., AP, acute pancreatitis; SAP, severe acute pancreatitis;RT, routine treatment; E, experimental group; C, control group; D, Duration; F, Frequencies; d, day; bid, twice a day. 2004, Guidelines for diagnosis and Treatment of Acute Pancreatitis in China (Draft), 2004. 2014, Guidelines for the diagnosis and treatment of acute pancreatitis (2014 edition) -Chinese Medical Association. ①: Total effectiveness rate ②: Time until relief of abdominal pain ③: Time until relief of abdominal distension ④: Serum amylase level ⑤: White blood cell recovery time ⑥: Hospital stays ⑦: IL—6 level ⑧: IL—8 level ⑨: TNF-α level ⑩: CRP.

### 3.3 Methodological assessment

The AMSTAR-2 was used to evaluate the methodological quality of the literature, among which Items 2, 4, 7, 9, 11, 13, and 15 are critical items. The evaluation results showed that the methodological quality of eight SRs was rated as critically low, and one SR was rated as low. For the content of different items, we summarized the advantages and disadvantages of all articles. The advantages included the following aspects: The research questions and inclusion criteria for the SRs based on PICO principle (Item 1): The data extraction was repeatable, meaning that this work was done independently by two reviewers [Item 5 and Item 6, except (Dong and Huang, 2011; Jie et al., 2014; Chen et al., 2021a)]; The researchers used appropriate statistical analysis methods to synthesize the results (Item 11); The risk of bias in individual studies was considered in interpreting and discussing the results of the SRs [Item 13, except (Dong and Huang, 2011; Jie et al., 2014)]. The shortcomings were also obvious. Most of the SRs had no preliminary research plans (Item 2), the selection basis of the research design had no reasonable explanation (Item 3), the list of excluded studies and reasons were not provided (Item 7), and the potential sources of conflicts of interest were not reported (Item 16). Other items were only reported in partial SRs. The detailed results are shown in Table 2.

**TABLE 2:** Methodological quality assessment of SRs by AMSTAR-2.

AMSTAR-2	Chen et al. (2021a)	Xu et al. (2021)	Zhou et al. (2021)	Ma et al. (2017)	Zhang and Ke. (2016)	Zheng et al. (2015)	Jie R. et al. (2014)	Dond and Huang. (2011)	Li et al. (2010)	Yes [n (%)]
Item 1	Y	Y	Y	Y	Y	Y	Y	Y	Y	9 (100)
**Item 2**	**Y**	**N**	**N**	**N**	**N**	**N**	**N**	**N**	**N**	**1(11.11)**
Item 3	N	N	N	N	N	N	N	N	N	0 (0)
**Item 4**	**PY**	**PY**	**PY**	**PY**	**PY**	**PY**	**PY**	**PY**	**PY**	**0(0)**
Item 5	N	Y	Y	N	Y	N	N	N	Y	4 (44.44)
Item 6	N	Y	Y	Y	Y	Y	N	N	Y	6 (66.66)
**Item 7**	**Y**	**N**	**N**	**N**	**N**	**N**	**N**	**N**	**N**	**1(11.11)**
Item 8	Y	Y	PY	PY	PY	PY	PY	PY	PY	2 (22.22)
**Item 9**	**Y**	**Y**	**Y**	**N**	**Y**	**PY**	**Y**	**N**	**Y**	**6(66.66)**
Item 10	N	N	N	N	N	Y	N	N	N	1 (11.11)
**Item 11**	**Y**	**Y**	**Y**	**Y**	**Y**	**Y**	**Y**	**Y**	**Y**	**9(100)**
Item 12	Y	Y	Y	N	Y	N	N	N	N	4 (44.44)
**Item 13**	**Y**	**Y**	**Y**	**Y**	**Y**	**Y**	**N**	**N**	**Y**	**7(77.77)**
Item 14	Y	Y	Y	N	N	Y	N	N	Y	5 (55.55)
**Item 15**	**Y**	**Y**	**Y**	**N**	**Y**	**N**	**Y**	**N**	**N**	**5(55.55)**
Item 16	Y	Y	N	N	N	N	N	N	N	2 (22.22)
Ranking of quality	Low	Critically low	Critically low	Critically low	Critically low	Critically low	Critically low	Critically low	Critically low	

Y, yes; PY, partial yes; N, no; SRs, systematic reviews; Bold values are critical items of AMSTAR-2.

### 3.4 Reporting quality

Among them, Item 1 (Title), Item 3 (Rationale), Item 4 (Objectives), Item 6 (Eligibility criteria), Item 9 (Study selection), Item 13 (Summary measures), Item 14 (Synthesis of results), Item 20 (Results of individual studies), Item 21 (Synthesis of results), Item 25 (Limitations), and Item 26 (Conclusions) have received detailed reports, but the condition of Item 2 (Structured summary), Item 5 (Protocol and registration), Item 11 (Data items), and Item 27 (Funding) in the report is incomplete (<30%). Some of the SRs reported the contents of other items. The detailed results are shown in Table 3.

**TABLE 3:** Reporting quality assessment of SRs by PRISMA checklist.

Section/topic	Items	Included studies									
		Chen et al. (2021a)	Xu et al. (2021)	Zhou et al. (2021)	Ma et al. (2017)	Zhang and Ke. (2016)	Zheng et al. (2015)	Ren et al. (2014)	Dond and Huang. (2011)	Li et al. (2010)	Yes [n (%)]
Title	Item 1	Y	Y	Y	Y	Y	Y	Y	Y	Y	9 (100)
Abstract	Item 2	Y	PY	PY	PY	PY	PY	PY	PY	PY	1 (11.11)
Introduction	Item 3	Y	Y	Y	Y	Y	Y	Y	Y	Y	9 (100)
	Item 4	Y	Y	Y	Y	Y	Y	Y	Y	Y	9 (100)
Methods	Item 5	Y	N	N	N	N	N	N	N	N	1 (11.11)
	Item 6	Y	Y	Y	Y	Y	PY	Y	Y	PY	7 (77.77)
	Item 7	Y	Y	PY	PY	Y	PY	PY	PY	Y	4 (44.44)
	Item 8	Y	N	Y	N	N	N	N	N	N	2 (22.22)
	Item 9	Y	Y	Y	Y	N	Y	N	Y	Y	7 (77.77)
	Item 10	Y	Y	Y	PY	Y	PY	N	N	Y	5 (55.55)
	Item 11	Y	Y	PY	PY	PY	PY	PY	PY	PY	2 (22.22)
	Item 12	Y	Y	Y	N	Y	N	Y	N	N	5 (55.55)
	Item 13	Y	Y	Y	Y	Y	Y	Y	Y	Y	9 (100)
	Item 14	Y	Y	Y	Y	Y	Y	Y	Y	Y	9 (100)
	Item 15	Y	Y	Y	N	Y	N	Y	N	N	5 (55.55)
	Item 16	Y	N	Y	Y	N	Y	N	N	Y	5 (55.55)
Results	Item 17	Y	Y	Y	PY	N	PY	N	PY	PY	3 (33.33)
	Item 18	Y	Y	Y	Y	Y	Y	Y	Y	PY	8 (88.88)
	Item 19	Y	Y	Y	N	Y	N	N	N	N	4 (44.44)
	Item 20	Y	Y	Y	Y	Y	Y	Y	Y	Y	9 (100)
	Item 21	Y	Y	Y	Y	Y	Y	Y	Y	Y	9 (100)
	Item 22	Y	Y	Y	N	Y	N	Y	N	N	5 (55.55)
	Item 23	Y	N	Y	Y	Y	N	N	N	N	4 (44.44)
Discussion	Item 24	Y	Y	PY	PY	Y	PY	PY	PY	Y	4 (44.44)
	Item 25	Y	Y	Y	Y	Y	Y	Y	PY	Y	8 (88.88)
	Item 26	Y	Y	Y	Y	Y	Y	PY	Y	Y	8 (88.88)
Funding	Item 27	Y	Y	N	N	N	PY	N	N	N	2 (22.22)

Y, yes; PY, partial yes; N, no; SRs, systematic reviews.

### 3.5 Quality of evidence

A total of 47 results were included in the nine SRs, of which five results were moderate quality, 22 results were low quality and 20 results were very low quality. RoB (47/47, 100%), inconsistency (31/47, 65.95%), and publication bias (31/47, 65.95%) were the main factors for demoting results. The detailed results are shown in Table 4.

**TABLE 4:** Quality of evidence in the included studies assessed by GRADE.

Author	Outcomes (participants)	Certainty assessment					Effect estimate (95% CI)	p Value	Quality of evidence
		Risk of bias	Inconsistency	Indirectness	Imprecision	Publication bias			
Chen et al. (2021a)	Total effectiveness rate (1882)	Serious^a^	Not serious	Not serious	Not serious	Not serious	RR 1.16 (1.12, 1.20)	<0.00001	Moderate
	Time until relief of abdominal pain (1,093)	Serious^a^	Serious^b^	Not serious	Not serious	Not serious	MD −1.74 (−1.96, −1.52)	<0.00001	Low
	Time until relief of abdominal distension (637)	Serious^a^	Serious^b^	Not serious	Not serious	Not serious	MD −1.56 (−2.07, −1.04)	<0.00001	Low
	Serum amylase level (508)	Serious^a^	Serious^b^	Not serious	Not serious	Not serious	MD −105.61 (−173.77, −37.46)	0.002	Low
	White blood cell recovery time (586)	Serious^a^	Serious^b^	Not serious	Not serious	Not serious	MD −1.51 (−1.66, −1.36)	<0.00001	Low
	IL—6 level (984)	Serious^a^	Serious^b^	Not serious	Not serious	Not serious	MD -18.22 (-23.36, -13.08)	<0.00001	Low
	CRP (560)	Serious^a^	Serious^b^	Not serious	Not serious	Not serious	MD −11.05 (−14.32, −7.78)	<0.00001	Low
Xu et al. (2021)	Total effectiveness rate (3,297)	Serious^a^	Not serious	Not serious	Not serious	Serious^d^	RR 1.20 (1.17, 1.24)	<0.00001	Very low
	Time until relief of abdominal pain (2,312)	Serious^a^	Serious^b^	Not serious	Not serious	Serious^d^	MD −1.64 (−1.87, −1.40)	<0.00001	Very low
	Time until relief of abdominal distension (1,600)	Serious^a^	Serious^b^	Not serious	Not serious	Serious^d^	MD −1.48 (−1.78, −1.18)	<0.00001	Very low
	Serum amylase level (1,084)	Serious^a^	Serious^b^	Not serious	Not serious	Serious^d^	MD −112.45 (−152.24, −72.67)	<0.00001	Very low
	White blood cell recovery time (931)	Serious^a^	Serious^b^	Not serious	Not serious	Serious^d^	MD −2.44 (−3.13, −1.76)	<0.00001	Very low
	Hospital stays (569)	Serious^a^	Serious^b^	Not serious	Not serious	Serious^d^	MD −4.84 (−6.78, −2.90)	<0.00001	Very low
	IL—6 level (2,383)	Serious^a^	Serious^b^	Not serious	Not serious	Serious^d^	SMD −2.11 (−2.54, −1.69)	<0.00001	Very low
	IL—8 level (1,162)	Serious^a^	Serious^b^	Not serious	Not serious	Serious^d^	SMD −1.98 (−2.57, −1.40)	<0.00001	Very low
	TNF-α level (2,783)	Serious^a^	Serious^b^	Not serious	Not serious	Serious^d^	SMD −2.06 (−2.43, −1.68)	<0.00001	Very low
	CRP (2,594)	Serious^a^	Serious^b^	Not serious	Not serious	Serious^d^	MD −2.12 (−2.51, −1.72)	<0.00001	Very low
Zhou et al. (2021)	Total effectiveness rate (364)	Serious^a^	Not serious	Not serious	Not serious	Serious^d^	RR 1.19 (1.09, 1.29)	<0.0001	Low
	Time until relief of abdominal pain (411)	Serious^a^	Not serious	Not serious	Not serious	Serious^d^	MD −1.65 (−2.03, −1.27)	<0.00001	Low
	Time until relief of abdominal distension (316)	Serious^a^	Not serious	Not serious	Not serious	Serious^d^	MD −1.29 (−1.75, −0.82)	<0.00001	Low
	Serum amylase level (411)	Serious^a^	Not serious	Not serious	Not serious	Serious^d^	MD −1.58 (−2.32, −0.84)	<0.0001	Low
	White blood cell recovery time (411)	Serious^a^	Not serious	Not serious	Not serious	Serious^d^	MD −2.62 (−3.08, −2.16)	<0.00001	Low
	TNF-α level (522)	Serious^a^	Not serious	Not serious	Not serious	Not serious	SMD −2.02 (−2.61, −1.43)	<0.00001	Moderate
	IL-6 level (584)	Serious^a^	Not serious	Not serious	Not serious	Not serious	SMD −1.59 (−2.11, −1.06)	<0.00001	Moderate
Ma et al. (2017)	Total effectiveness rate (326)	Serious^a^	Not serious	Not serious	Not serious	Serious^d^	RR 1.16 (1.07, 1.25)	0.0002	Low
	Time until relief of abdominal pain (813)	Serious^a^	Serious^b^	Not serious	Not serious	Not serious	MD −1.71 (−2.21, −1.21)	<0.01	Low
	Serum amylase level (813)	Serious^a^	Serious^b^	Not serious	Not serious	Not serious	MD −1.82 (−2.39, −1.25)	<0.01	Low
	White blood cell recovery time (553)	Serious^a^	Not serious	Not serious	Not serious	Not serious	MD −2.75 (−3.19, −2.31)	<0.01	Moderate
	Hospital stays (55)	Serious^a^	Serious^b^	Not serious	Not serious	Not serious	MD −5.99 (−7.73, −4.26)	<0.01	Low
	IL-6 level (226)	Serious^a^	Serious^b^	Not serious	Not serious	Serious^d^	SMD −1.09 (−2.66, 0.48)	<0.01	Very low
	IL-8 level (212)	Serious^a^	Serious^b^	Not serious	Not serious	Serious^d^	SMD −1.02 (−1.66, −0.38)	<0.01	Very low
	TNF-α level (226)	Serious^a^	Serious^b^	Not serious	Not serious	Serious^d^	SMD −1.10 (−1.68, −0.53)	<0.01	Very low
Zhang and Ke. (2016)	IL-6 level (616)	Serious^a^	Serious^b^	Not serious	Not serious	Not serious	SMD 1.86 (1.46, 2.27)	<0.001	Low
	IL-8 level (397)	Serious^a^	Serious^b^	Not serious	Not serious	Serious^d^	SMD 2.16 (1.45, 2.88)	<0.001	Very low
	TNF-α level (760)	Serious^a^	Serious^b^	Not serious	Not serious	Not serious	SMD 2.16 (1.56, 2.76)	<0.001	Low
Zheng et al. (2015)	Total effectiveness rate (585)	Serious^a^	Not serious	Not serious	Not serious	Not serious	RR 0.85 (0.80, 0.91)	<0.00001	Moderate
	Hospital stays (280)	Serious^a^	Serious^b^	Not serious	Not serious	Serious^d^	MD −5.28 (−6.69, −3.86)	<0.00001	Very low
Ren et al. (2014)	Total effectiveness rate (326)	Serious^a^	Not serious	Not serious	Not serious	Serious^d^	RR 1.16 (1.07, 1.25)	0.0002	Low
	Time until relief of abdominal pain (323)	Serious^a^	Serious^b^	Not serious	Not serious	Serious^d^	MD −1.86 (−2.27, −1.45)	<0.00001	Very low
	Serum amylase level (421)	Serious^a^	Serious^b^	Not serious	Not serious	Serious^d^	MD −1.80 (−2.68, −0.93)	<0.00001	Very low
	White blood cell recovery time (245)	Serious^a^	Serious^b^	Not serious	Not serious	Serious^d^	MD −2.49 (−3.56, −1.41)	<0.00001	Very low
Dond and Huang. (2011)	Time until relief of abdominal pain (513)	Serious^a^	Serious^b^	Not serious	Not serious	Not serious	MD −1.80 (−2.21, −1.38)	<0.00001	Low
	Serum amylase level (297)	Serious^a^	Serious^b^	Not serious	Not serious	Serious^d^	MD −2.61 (−3.01, −2.20)	<0.00001	Very low
	Hospital stays (303)	Serious^a^	Serious^b^	Not serious	Serious^c^	Serious^d^	MD −4.66 (−7.02, −2.31)	<0.00001	Very low
Li et al. (2010)	Time until relief of abdominal pain (197)	Serious^a^	Not serious	Not serious	Not serious	Serious^d^	MD −1.91 (−2.19, −1.63)	<0.00001	Low
	Time until relief of abdominal distension (197)	Serious^a^	Not serious	Not serious	Not serious	Serious^d^	MD −1.88 (−2.33, −1.43)	<0.00001	Low
	Serum amylase level (242)	Serious^a^	Not serious	Not serious	Not serious	Serious^d^	MD −2.58 (−3.02, −2.15)	<0.00001	Low
	White blood cell recovery time (242)	Serious^a^	Not serious	Not serious	Not serious	Serious^d^	MD −1.78 (−2.24, −1.32)	<0.00001	Low

GRADE, Grading of Recommendations Assessment, Development, and Evaluation.

a The risk of bias is unclear in most of the studies.

b The confidence interval overlap less, the heterogeneity test P was very small, and the I2 was larger (I^2^ threshold value: 50%).

c The sample size is small, and the CI, is wide.

d Funnel plot was not symmetrical, or the number of included studies was small and all were positive results (sample size threshold value: 500).

### 3.6 Descriptive synthesis and risk of bias

We performed a descriptive synthesis of ten outcomes, including total effectiveness rate, time until relief of abdominal pain, time until relief of abdominal distension, serum amylase level, white blood cell recovery time, hospital stay, IL-6 level, IL-8 level, TNF-α level, and CRP. At least two SRs evaluated these outcomes. The results showed that compared with the control group, the Xuebijing injection combined treatment group could improve the total effectiveness rate better in six SRs (Jie et al., 2014; Zheng et al., 2015; Ma et al., 2017; Chen et al., 2021a; Xu et al., 2021; Zhou et al., 2021). Seven SRs (Li et al., 2010; Dong and Huang, 2011; Jie et al., 2014; Ma et al., 2017; Chen et al., 2021a; Xu et al., 2021; Zhou et al., 2021) and four SRs (Li et al., 2010; Chen et al., 2021a; Xu et al., 2021; Zhou et al., 2021) reduced the relief time of abdominal pain and abdominal distention in AP patients, respectively. Seven SRs (Li et al., 2010; Dong and Huang, 2011; Jie et al., 2014; Ma et al., 2017; Chen et al., 2021a; Xu et al., 2021; Zhou et al., 2021) significantly reduced serum amylase levels. Other outcomes also showed significant differences compared with the control group as detailed in Table 4.

**FIGURE 2:**
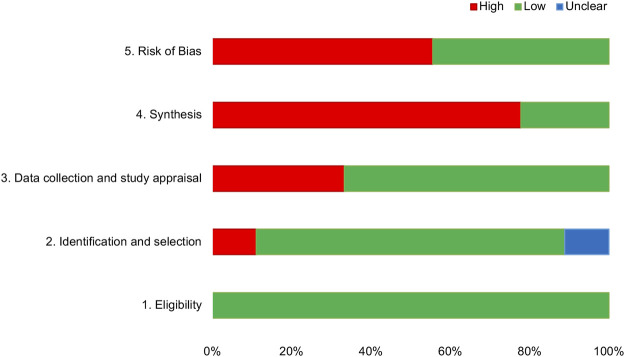
Graphical presentation of risk of bias of the included SRs.

The RoB assessments of SRs included in this overview are shown in Table 5 and Figure 2. Five SRs (55.55%) were rated as low risk, and four SRs (44.44%) were rated as high risk through the comprehensive assessment in phase 3. Failure to properly explain and deal with the RoB may lead to high RoB in SR.

**TABLE 5:** Tabular presentation for ROBIS results.

Review		Phase 2		Phase 3	
	Study eligibility criteria	Identification and selection of studies	Data collection and study appraisal	Synthesis and findings	Risk of bias in the review
Chen et al. (2021a)	☺	☺	☹	☺	☺
Xu et al. (2021)	☺	?	☺	☹	☹
Zhou et al. (2021)	☺	☺	☺	☺	☺
Ma et al. (2017)	☺	☹	☺	☹	☹
Zhang and Ke. (2016)	☺	☺	☺	☹	☺
Zheng et al. (2015)	☺	☺	☺	☹	☺
Ren et al. (2014)	☺	☺	☹	☹	☹
Dond and Huang. (2011)	☺	☺	☹	☹	☹
Li et al. (2010)	☺	☺	☺	☹	☺

☺, low risk; ☹, high risk; ?, unclear risk.

### 3.7 Results of the data synthesis and quantitative analysis

#### 3.7.1 Total effectiveness rate

**FIGURE 3:**
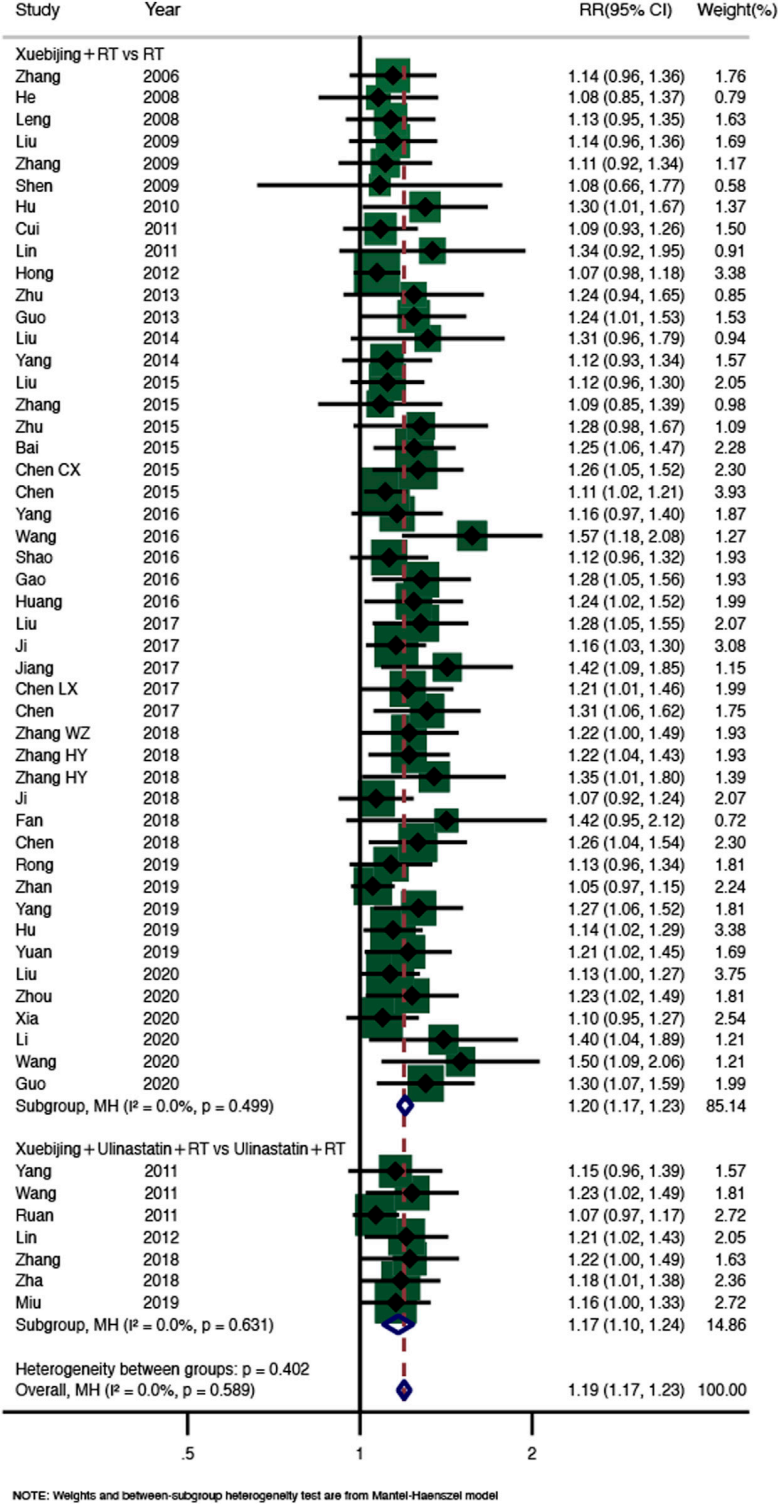
Meta-analysis of total effectiveness rate.

A total of six SRs reported the total effectiveness rate (Jie et al., 2014; Zheng et al., 2015; Ma et al., 2017; Chen et al., 2021a; Xu et al., 2021; Zhou et al., 2021). We conducted a meta-analysis again, and 54 RCTs (2,432 participants) were included after screening and eliminating duplicates. The results showed that Xuebijing injection combined treatment increased the total effectiveness rate of AP patients (RR = 1.19, 95% CI 1.17–1.23, *p* < 0.0001), and there was no heterogeneity between studies (I^2^ = 0.0%, *p* = 0.589). According to the different combinations, we divided them into two subgroups, and the results of subgroup analysis were consistent with the overall results (Xuebijing with RT group, RR = 1.20, 95% CI 1.17–1.23, *p* < 0.0001, 47 RCTs, 1,828 participants; Xuebijing with ulinastatin group, RR = 1.17, 95% CI 1.10–1.24, *p* < 0.0001, 7 RCTs, 604 participants). See Figure 3 for specific analysis results. A funnel plot and Egger’s test showed publication bias, and sensitivity analysis showed that the results were stable (Supplementary Figures S1–S3).

#### 3.7.2 Time until relief of abdominal pain

**FIGURE 4:**
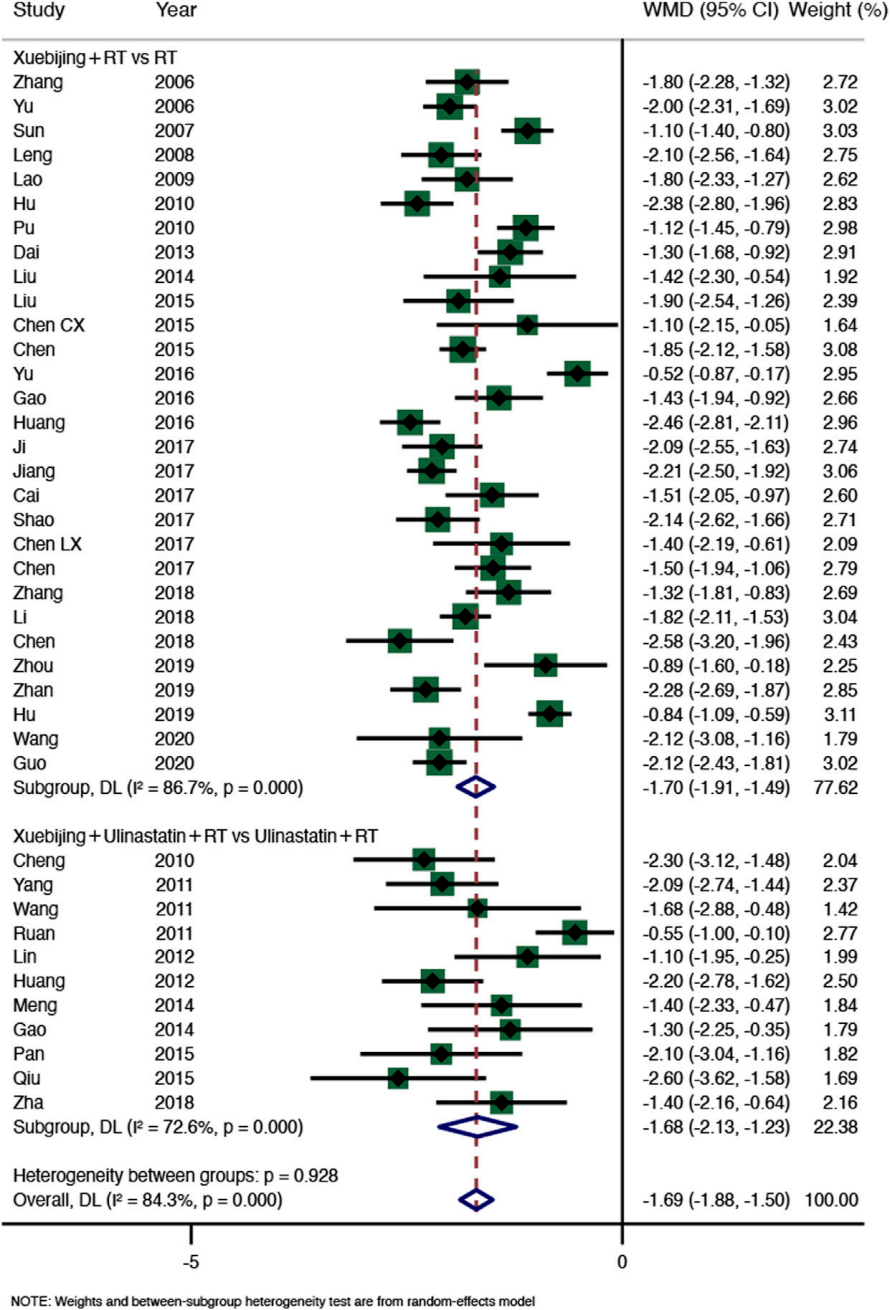
Meta-analysis of time until relief of abdominal pain.

Forty RCTs (3,328 participants) from seven SRs (Li et al., 2010; Dong and Huang, 2011; Jie et al., 2014; Ma et al., 2017; Chen et al., 2021a; Xu et al., 2021; Zhou et al., 2021) reported abdominal pain relief time (Figure 4). The results showed that compared with the control group, the Xuebijing injection combined treatment group shortened the abdominal pain relief time in AP patients (WMD = −1.69, 95% CI −1.88–−1.50, *p* < 0.0001), with high heterogeneity (I^2^ = 84.3%, *p* = 0.000), so the random-effect model was selected. The results of subgroup analysis showed that there were significant differences between the two subgroups compared with the control group, and the heterogeneity was still significant (Xuebijing with RT group, WMD = −1.70, 95% CI −1.91–−1.49, *p* < 0.0001, 29 RCTs, 2,417 participants; Xuebijing with ulinastatin group, WMD = −1.68, 95% CI −2.13–−1.23, *p* < 0.0001, 11 RCTs, 911 participants). Sensitivity analysis was used to further investigate the source of heterogeneity, and after successive exclusion of each study, none of the studies affected the pooled analysis (Supplementary Figure S4).

#### 3.7.3 Time until relief of abdominal distension

**FIGURE 5:**
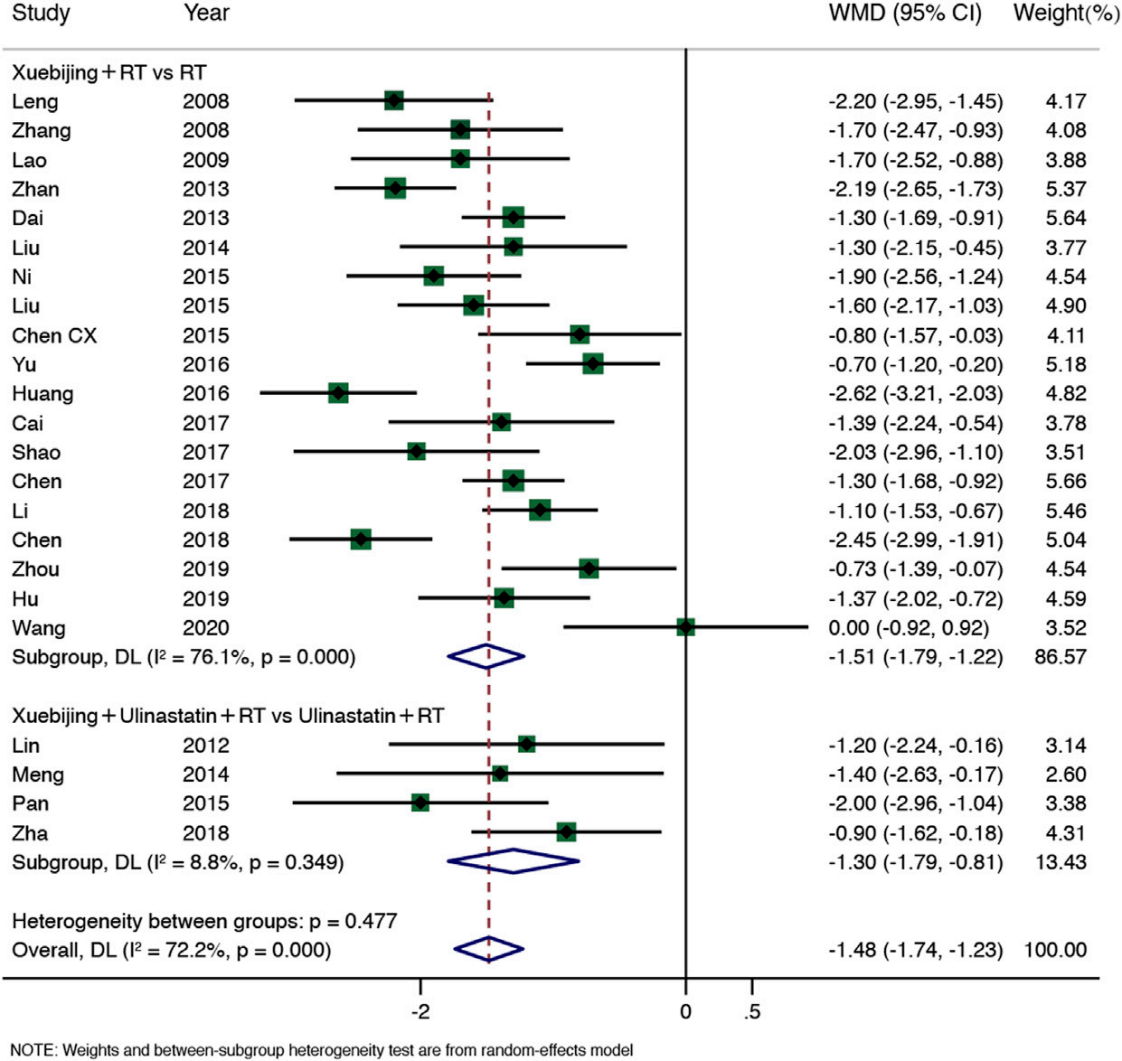
Meta-analysis of time until relief of abdominal distension.

Four SRs (Li et al., 2010; Chen et al., 2021a; Xu et al., 2021; Zhou et al., 2021) including 23 RCTs (1,842 participants) showed that Xuebijing injection combined treatment reduced abdominal distension relief time in AP patients (WMD = −1.48, 95% CI −1.74–−1.23, *p* < 0.0001), but the heterogeneity was high (I^2^ = 72.2%, *p* = 0.000), so the random-effect model was adopted. The results of subgroup 1 (Xuebijing with RT group: WMD = −1.51, 95% CI−1.79–−1.22, *p* < 0.0001, 19 RCTs, 1,526 participants) and subgroup 2 (Xuebijing with Ulinastatin group: WMD = −1.30, 95% CI −1.79–−0.81, *p* < 0.0001, 4 RCTs, 316 participants) were significantly different. However, subgroup 1 still had significant heterogeneity (I^2^ = 76.1%, *p* = 0.000), and subgroup 2 had no heterogeneity (I^2^ = 8.8%, *p* = 0.349) (Figure 5). No source of heterogeneity was found in the sensitivity analysis (Supplementary Figure S5).

#### 3.7.4 Serum amylase level

**FIGURE 6:**
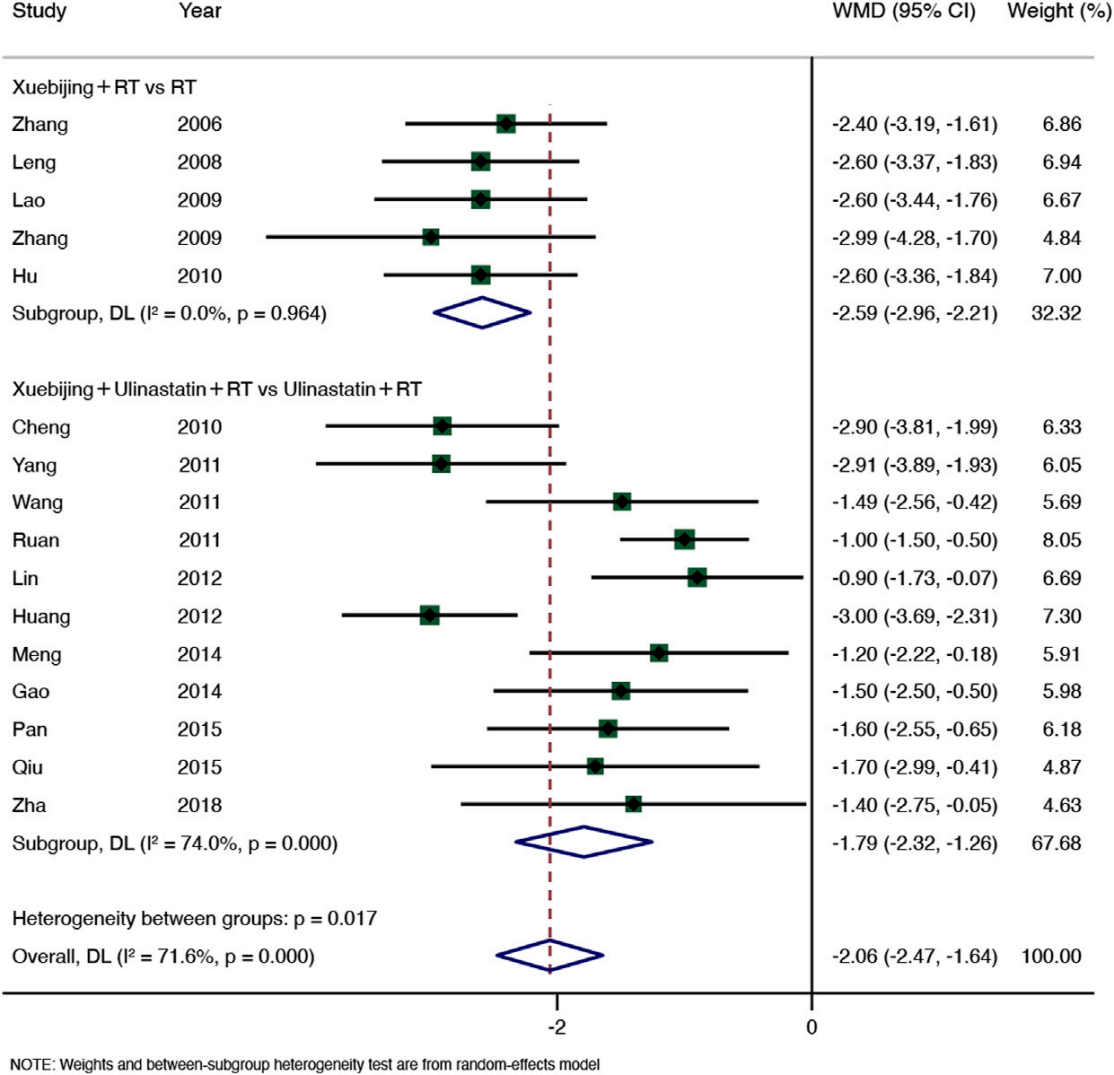
Meta-analysis of serum amylase level.

Sixteen RCTs (1,182 participants) of the seven SRs (Li et al., 2010; Dong and Huang, 2011; Jie et al., 2014; Ma et al., 2017; Chen et al., 2021a; Xu et al., 2021; Zhou et al., 2021) showed that Xuebijing injection combined treatment could reduce serum amylase levels (WMD = −2.06, 95% CI −2.47–−1.64, *p* < 0.0001), with significant heterogeneity (I^2^ = 71.6%, *p* = 0.000), so the random-effect model was adopted. The results of subgroup 1 (Xuebijing with RT group: WMD = −2.59, 95% CI −2.96–−2.21, *p* < 0.0001, 5 RCTs, 307 participants) and subgroup 2 (Xuebijing with Ulinastatin group: WMD = −1.79, 95% CI −2.32–−1.26, *p* < 0.0001, 11 RCTs, 875 participants) were significantly different. However, subgroup 1 had no heterogeneity (I^2^ = 0.0%, *p* = 0.964), and subgroup 2 still had high heterogeneity (I^2^ = 74%, *p* = 0.000) (Figure 6). Further sensitivity analysis failed to find the source of heterogeneity (Supplementary Figure S6).

#### 3.7.5 Subgroup analysis

In addition to subgroup analysis based on intervention measures, we also conducted a subgroup analysis according to the degree of patients’ condition (AP and SAP). Meta-analysis showed that compared with the control group, Xuebijing had certain advantages in improving the total effectiveness rate, reducing serum amylase level, and shortening the remission time of abdominal pain in patients with AP and SAP, but had no significant advantage in shortening the remission time of abdominal distension in AP patients (*p* = 0.291). Meanwhile, subgroup analysis showed low heterogeneity in the total effectiveness rate of AP/SAP and serum amylase levels of AP, other subgroups still had significant heterogeneity, and sensitivity analysis showed that the results are stable (Supplementary Figures S7–S10).

### 3.8 Adverse drug reaction

A total of one SR reported ADRs. Zheng (Zheng et al., 2015) noted that in the Xuebijing group, one patient had headache and nausea and a slight rash, which disappeared spontaneously after drug withdrawal. No ADRs were observed in the control group.

## 4 Discussion

### 4.1 Summary of key findings

This overview comprehensively evaluated the methodological and reporting quality of nine SRs on the efficacy and safety of Xuebijing injection in the treatment of AP/SAP. A single SR seems to suggest the benefit of Xuebijing injection in improving AP/SAP. In the PRISMA checklist, the quality of SRs was relatively good, and the manuscript structures were relatively complete. However, the methodological quality of SR was low (1/9) or critically low (8/9). Because the seven SRs were all published before 2017, the AMSTAR-2 tool we used (updated in 2017) may have a certain bias in the methodological quality assessment results of SRs. In addition, more than 80% of GRADE evidence was rated as low or very low, with five SRs rated as low risk of bias and four SRs rated as high risk of bias. Finally, the RCTs involved in SRs were summarized and quantitatively analyzed. The results showed that Xuebijing injection improved the total effectiveness rate in AP/SAP patients. The time until relief of abdominal pain and time until relief of abdominal distension in AP patients were reduced, and the serum amylase level was decreased. Although there was significant heterogeneity in some outcomes, in general, we still support that Xuebijing injection combined treatment is effective in the treatment of AP/SAP.

### 4.2 Discussion of systematic reviews evaluation results

The evaluation results of different SRs showed the positive effect of Xuebijing injection on AP/SAP, but we also found some problems: 1) Differences in diagnostic criteria, intervention, outcomes and evaluation time points of original RCTs lead to varying degrees of risk of bias in RCTs, and safety indicators are often ignored, thus increasing the risk of bias in SRs. 2) The authors of SRs did not standardize the legal contents of the other side, such as no preliminary research protocol, no specific exclusion list, and no reasonable use of the risk of bias assessment tool. Simultaneously, the authors did not further explain the source of heterogeneity and the risk of bias, which to some extent reduced the reliability of the effectiveness and safety of Xuebijing injection for AP/SAP.

Therefore, we have some suggestions based on existing problems. First, we identified high-quality RCTs according to the CONSORT statement (Schulz et al., 2010), optimized the study design, adopted internationally agreed diagnostic criteria and outcomes to reduce publication bias, and followed the PRISMA checklist for SR (Moher et al., 2009). The PICOS (population, interventions, outcomes, and study designs) framework should be used to build a clear and specific scientific problem. Changes in diagnostic criteria may affect different clinical outcomes and increase the heterogeneity of results. If necessary, subgroup analysis and sensitivity analysis should be performed, and the effect size can also be modified by trim and fill (Sutton et al., 2000). Second, the sources, dosage and frequency of Xuebijing injection, and ulinastatin should be reported as much as possible; otherwise, the final conclusion may be uncertain. Finally, the selection of outcomes is important, as different outcomes may lead to different conclusions. SR emphasized that all important outcomes related to decision-making should be included rather than determined by the original RCTs. Researchers should define primary and secondary indicators according to their main purpose or importance (Chen et al., 2021b).

### 4.3 Understanding of Chinese and western medicine for xuebijing injection

Gastrointestinal motility disorders often occur in AP patients, especially SAP patients, and abdominal pain and abdominal distension are common clinical symptoms (Wang et al., 2003). Analgesia and anti-inflammatory drugs are mainly used in clinical treatment, and opioids and nonsteroidal drugs are mainly selected, but the evidence of drug efficacy and safety is limited. Xuebijing injection is composed of *Paeonia lactiflora* Pal. (Shaoyao), *Ligusticum chuanxiong* hort (Chuanxiong), *Salvia miltiorrhiza* Bunge, *Carthamus tinctorius* L. (Honghua), and *Angelica sinensis* (Danggui), of which *Paeonia lactiflora* Pal. (Shaoyao) can function in dispersing blood stasis and relieving pain, clearing heat and cooling blood; *Ligusticum chuanxiong* hort (Chuanxiong) can function in dispelling wind, relieving pain, promoting qi and activating blood circulation; *Salvia miltiorrhiza* Bunge (Danshen) can function in promoting blood circulation, removing blood stasis and relieving pain, cooling blood and eliminating carbuncles; *Carthamus tinctorius* L. (Honghua) can function in removing blood stasis and relieving pain, promoting blood circulation and dredging menstruation; *Angelica sinensis* (Danggui) can function in invigorating blood circulation, relaxing meridians and relieving pain. Modern pharmacological studies have shown that Xuebijing injection can resist oxidative stress and improve microcirculation and intestinal mucosal barrier functions (Teng et al., 2015), moreover, it can bidirectionally regulate the inflammatory response, such as reducing serum amylase, white blood cell count and TNF-α levels in SAP patients, upregulating the level of the anti-inflammatory factor IL-10, and blocking the cascade reaction (Li, 2021). Therefore, Xuebijing injection, as an analgesic and anti-inflammatory Chinese medicine preparation, can improve abdominal pain, abdominal distension and other symptoms caused by AP/SAP, as well as alleviate inflammatory reactions.

### 4.4 Understanding adverse drug reactions in xuebijing injection

With the widespread use of TCM injections in clinical treatment, ADRs have gradually become the focus of medical circles and the public. However, among all SRs, only one SR reported ADRs of Xuebijing injection. Previous studies have made clinical statistics on the safety of Xuebijing injection. The results of a real-world study based on the ADRs of Xuebijing injection in 93 hospitals (31,913 cases) showed that the incidence of ADRs was 0.3%, mainly including skin pruritus, rash, chest intensity, fever, and labored breathing. And the occurrence of ADRs was related to injection speed and irrigating syringe (Zheng et al., 2019). Another study showed that ADRs induced by Xuebijing injection mainly occurred in patients aged 50 to 60 and were most likely to occur within 30 min after administration, in which respiratory system, skin and accessory damage were more common (Nie et al., 2019). In a study analyzing ADRs collected from 2014 to 2019, Xuebijing injection ranked 15th in the number and proportion of ADR reports of 60 TCM injections, but there was no death. Meanwhile, adverse reactions to Xuebijing injection were correlated with vehicle type, dosage, age, and drug combination. Therefore, the researchers suggested that the injection frequency of TCM should be reduced in children and elderly patients, and the close observation and strict monitoring should be required during the first 7 days after receiving injections (Wang et al., 2019; Huang et al., 2021). Therefore, the safety of Xuebijing injection still needs to be treated with caution, and the clinical quality control and standardized use should be strengthened in the clinic to avoid or reduce the occurrence of ADRs as far as possible.

### 4.5 Strengths and limitations

In recent years, many SRs showing that Xuebijing injection can effectively treat AP/SAP have been published. We must note that the value of overview lies in demonstrating the evidential quality and reliability of the results of SRs by collecting, analyzing, and presenting the descriptive characteristics as well as the quantitative outcome data in SRs. It has important guiding significance for clinical decision making. To our knowledge, this is the first comprehensive assessment of different SRs through inclusive retrieval and using standard assessment tools. In this overview, a comprehensive literature search was conducted on seven databases to comprehensively evaluate the efficacy and safety of Xuebijing injection in the treatment of AP/SAP by collecting existing data on different SRs. The PRISMA Checklist, AMSTAR-2, RoB Tool, and GRADE were used to comprehensively assess the methodological quality, quality of literature reporting, risk of bias, and level of evidence for SRs. Second, literature retrieval, data extraction, and evaluation were performed independently by at least two reviewers. Besides, we extracted the relevant data of RCTs involved in nine SRs for data synthesis and quantitative analysis, which can intuitively understand the overall quality of SR and the reliability of the results. Finally, this overview points out the existing problems and gives some reasonable suggestions for future research.

However, overview could only synthesize and describe all the data quantitatively. Differences in RCT study designs and the interventions may result in high RoB for SRs, which reduces the quality of the evidence and the methodology. Organ failure, local complications, and mortality are important endpoints of AP/SAP, which have not been reported in the SRs. Furthermore, we did not obtain relevant data from the original RCTs. Therefore, the original study should pay more attention to the effect of Xuebijing injection on long-term follow-up results, mortality and other endpoint events of AP/SAP. Finally, we must point out that there may be some deviations in our understanding of the assessment tools due to subjective factors, but we have tried to minimize the errors in the evaluation results.

## 5 Conclusion

After quantitative synthesis and analysis of the data in this study, we still support the clinical value of Xuebijing injection as an analgesic and anti-inflammatory Chinese medicine preparation in the treatment of AP/SAP. but the reliability of the results needs to be improved. The impact of Xuebijing injection on the endpoint events of AP/SAP is our focus in the future. High quality and large sample RCTs are still the key to obtain reliable clinical evidence. Clearly, high-quality, large-sample RCTs remain key to obtaining reliable clinical evidence.

## Data Availability

The original contributions presented in the study are included in the article/Supplementary Material, further inquiries can be directed to the corresponding authors.
