# Supplementary material for: Chinese herbal medicine xuebijing injection for acute pancreatitis: An overview of systematic reviews
Source: Front Pharmacol. 2022 Aug 10;13:883729. doi: 10.3389/fphar.2022.883729 (PMC9399720; doi:10.3389/fphar.2022.883729)
Supplement: Supplementary file 1 [file DataSheet2.PDF]

Supplementary tables

Search strategy sample of PubMed

#1	Pancreatitis [MeSH terms]
#2	Pancreatitis, Acute Hemorrhagic [MeSH terms]
#3	acute pancreatitis [text word]
#4	Or/#1-3
#5	xuebijing injection [text word]
#6	xuebijing [text word]
#7	Or/#5-6
#8	systematic review [text word]
#9	systematic evaluation [text word]
#10	meta-analysis [text word]
#11	Or/#8-10
#12	#4 and #7 and #11
((((systematic evaluation) OR (systematic review)) OR (meta-analysis)) AND ((xuebijing injection) OR (xuebijing))) AND (((Pancreatitis [MeSH Terms]) OR (Pancreatitis, Acute Hemorrhagic [MeSH Terms])) OR (acute pancreatitis))	

Search strategy sample of CNKI

SU=('血必净'+'血必净注射液') AND SU=('胰腺炎'+'急性胰腺炎') AND SU=('系统评价'+'meta 分析')

SU: subject search.

Cochrane detabase

#1 (xue-bi-jing):ti,ab,kw

#2 (xue bi jing):ti,ab,kw

#3 (xuebijing):ti,ab,kw

#4 (xuebijing injection):ti,ab,kw

#5 #1 OR #2 #3 #4

#6 (acute pancreatitis):ti,ab,kw

#7 (pancreatitis):ti,ab,kw

#8 (pancreatitis, acute hemorrhagic):ti,ab,kw

#9 #6 OR #7 OR #8

#10 (systematic evaluation):ti,ab,kw

#11 (systematic review):ti,ab,kw

#12 (meta-analysis):ti,ab,kw

#13 (meta):ti,ab,kw

#14 #10 OR #11 OR #12 OR #13

#21 #5 AND #9 AND #14

Embase detabase

#1 'acute pancreatitis':ti,ab,kw OR 'pancreatitis, acute hemorrhagic':ti,ab,kw OR ' pancreatitis '

#2 'injection':ti,ab,kw OR 'xuebijing':ti,ab,kw OR 'xuebijing injection':ti,ab,kw

#3 'systematic evaluation':ti,ab,kw OR 'systematic review':ti,ab,kw OR 'meta-analysis ':ti,ab,kw

#4 #1 AND #2 AND #3

SinoMed detabase

#1 "急性胰腺炎"[常用字段:智能] OR "胰腺炎"[常用字段:智能]

#2 "血必净"[常用字段:智能] OR "血必净注射液"[常用字段:智能]

#3 " meta 分析"[常用字段:智能] OR "系统评价"[常用字段:智能]

#4 (#3) AND (#2) AND (#1)

VIP

U=(急性胰腺炎+胰腺炎) AND U= (血必净+血必净注射液) AND U= (系统评价+ meta 分析)

Wanfang

主题:(“胰腺炎” or “急性胰腺炎”) and 主题: (“血必净” or “血必净注射液”) and 主题: (“meta 分析” or “系统评价”)

Excluded list.

Citation	Reason for exclusion
Tang Q, Tian L, Gao C, et al. The efficacy and safety of Xuebijing injection as an adjunctive treatment for acute pancreatitis: Protocol for a systematic review and meta-analysis of randomized controlled trials. Medicine (Baltimore). 2020 Jan;99(4): e18743.	Not SR
Li Bin, Xuebijing regional arterial perfusion in the treatment of severe acute pancreatitis: basic and clinical study. The First Hospital of Lanzhou University, Gansu Province,2018-07-23.	Not SR
Zinan Zhao, Bihua Zhang, Kexin Li, et al. Rapid hygienic technical evaluation of Xuebijing injection [J]. Exploration of Rational Drug Use in China,2020,17(09):6-14.	Not SR
Lu Hairu, Fan Lingqin. Evidence-based application, adverse reactions and precautions for use of Xuebijing injection[J]. Chinese Journal of New Drugs, 2013, 22(20): 2449-2452.	Not SR
Li Qing, Jin Xinyao, Zhou Xia, et al. The clinical safety evaluation of Xuebijing injection was differentiated among different research types [J]. Chinese Journal of Chinese Materia Medica, 2021, 46(03)):712-721.	Not SR
Sun Weihong, Ma Zetong, Chen Qian, et al. Meta analysis of adverse reactions and events of Xuebijing injection[J]. Journal of Ningxia Medical University, 2015, 37(11): 1296-1299.	Not acute pancreatitis

SR, systematic review
