# Supplementary material for: Chinese herbal medicine xuebijing injection for acute pancreatitis: An overview of systematic reviews
Source: Front Pharmacol. 2022 Aug 10;13:883729. doi: 10.3389/fphar.2022.883729 (PMC9399720; doi:10.3389/fphar.2022.883729)
Supplement: Supplementary file 2 [file DataSheet3.PDF]

Supplementary figures

Figure 1 Funnel plot of total effectiveness rate:

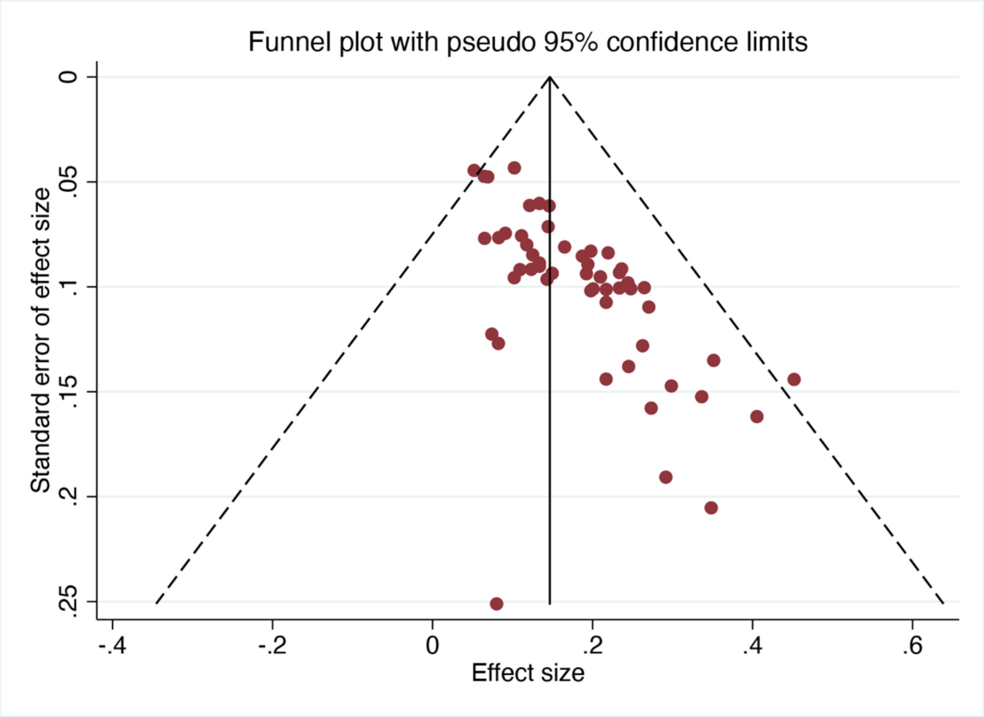

Figure 2 Egger's test and P value of total effectiveness rate:

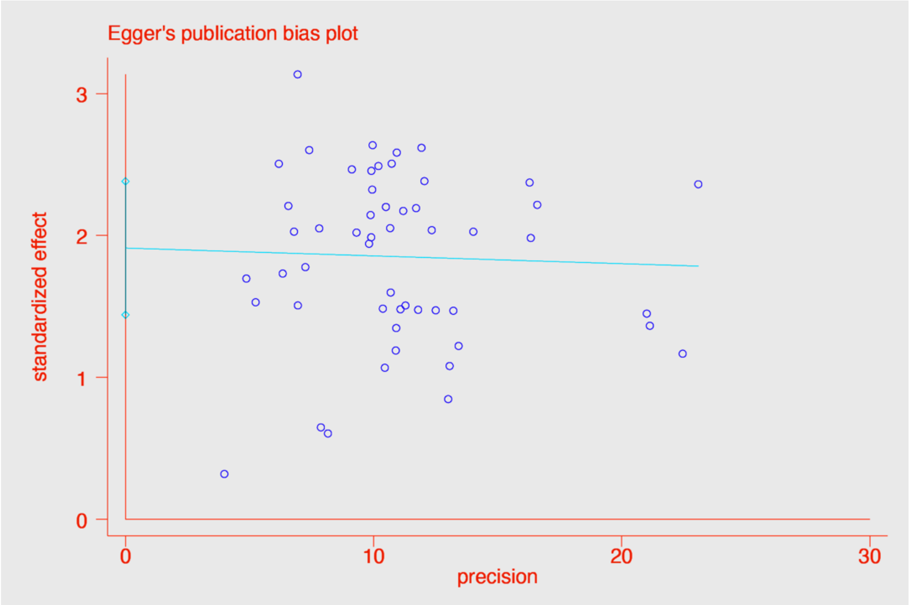

Egger's test

Std_Eff	Coef.	Std. Err.	t	P>|t|	[95% Conf.	Interval]
slope	-.0055024	.0198914	-0.28	0.783	-.0454174	.0344125
bias	1.911384	.2344032	8.15	0.000	1.441019	2.381748

Figure 3 Sensitivity analysis of total effectiveness rate:

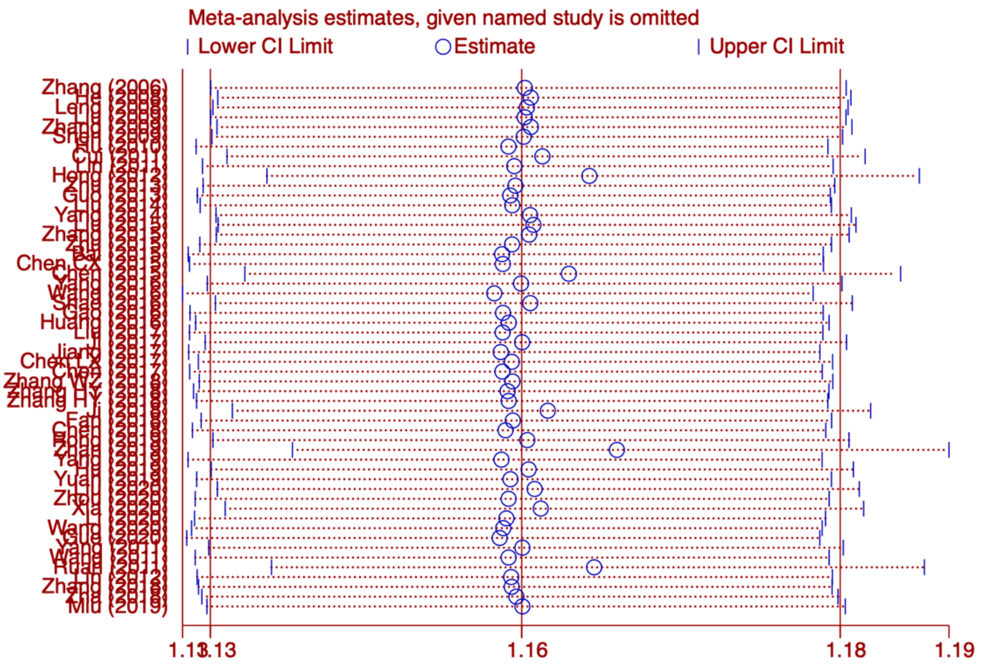

Figure 4 Sensitivity analysis of time until relief of abdominal pain:

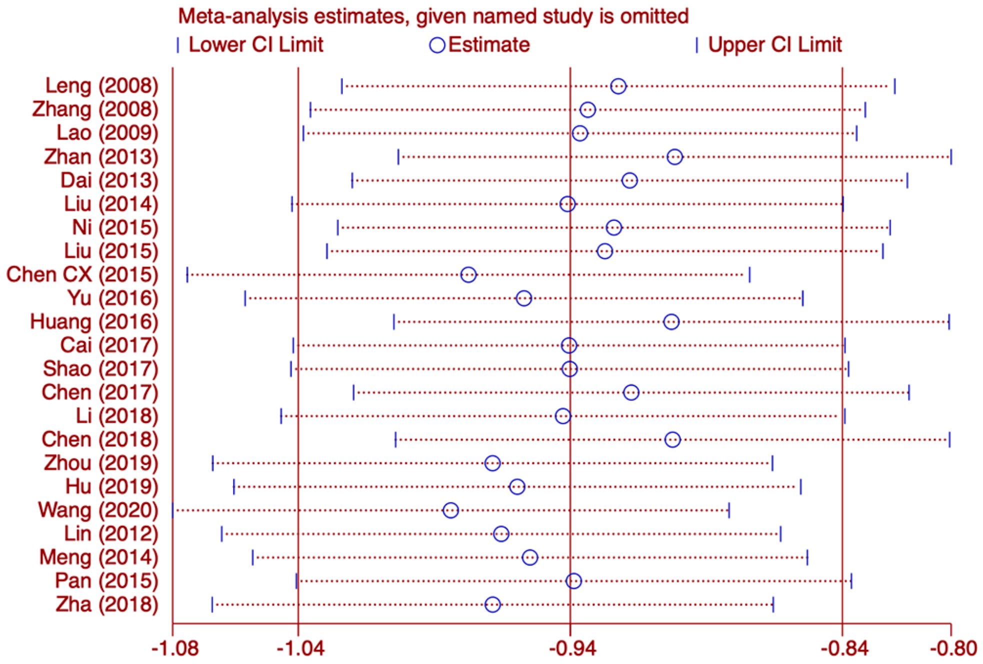

Figure 5 Sensitivity analysis of time until relief of abdominal distension:

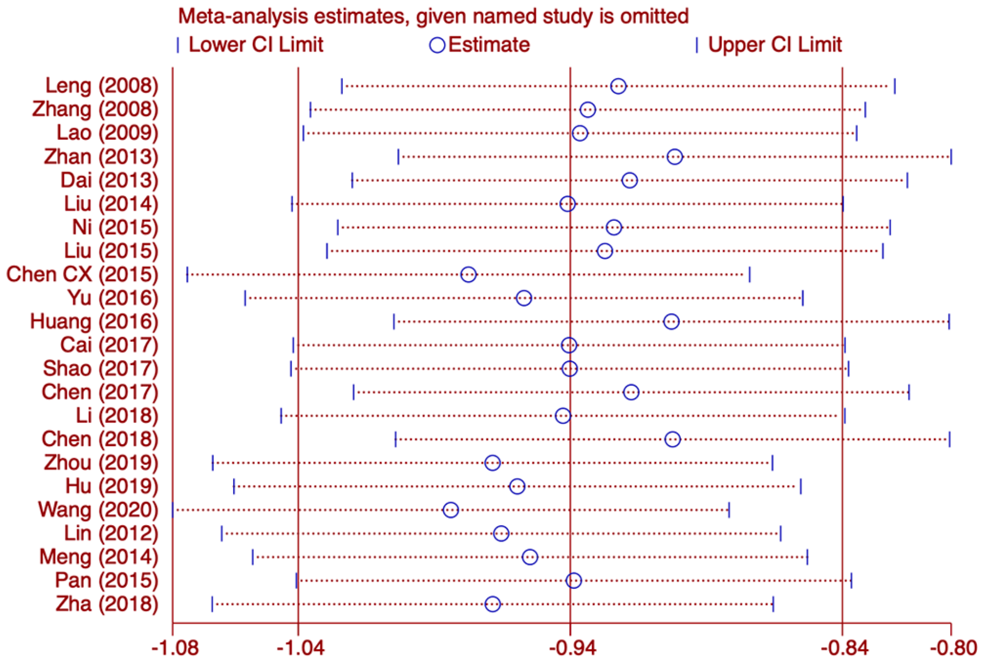

Figure 6 Sensitivity analysis of serum amylase level:

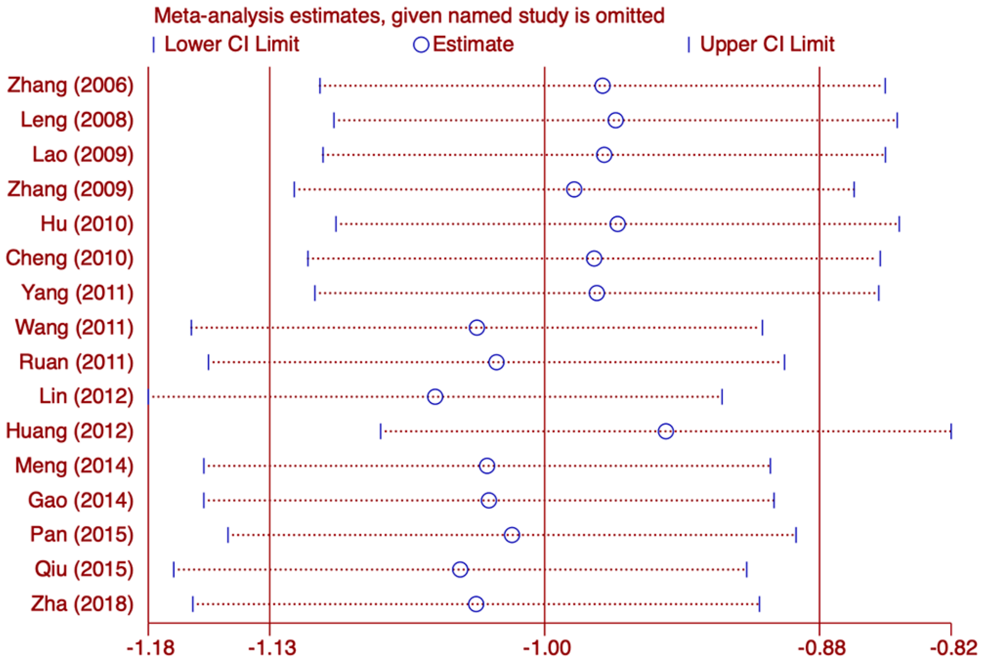

Subgroup analysis based on the degree of illness of patients

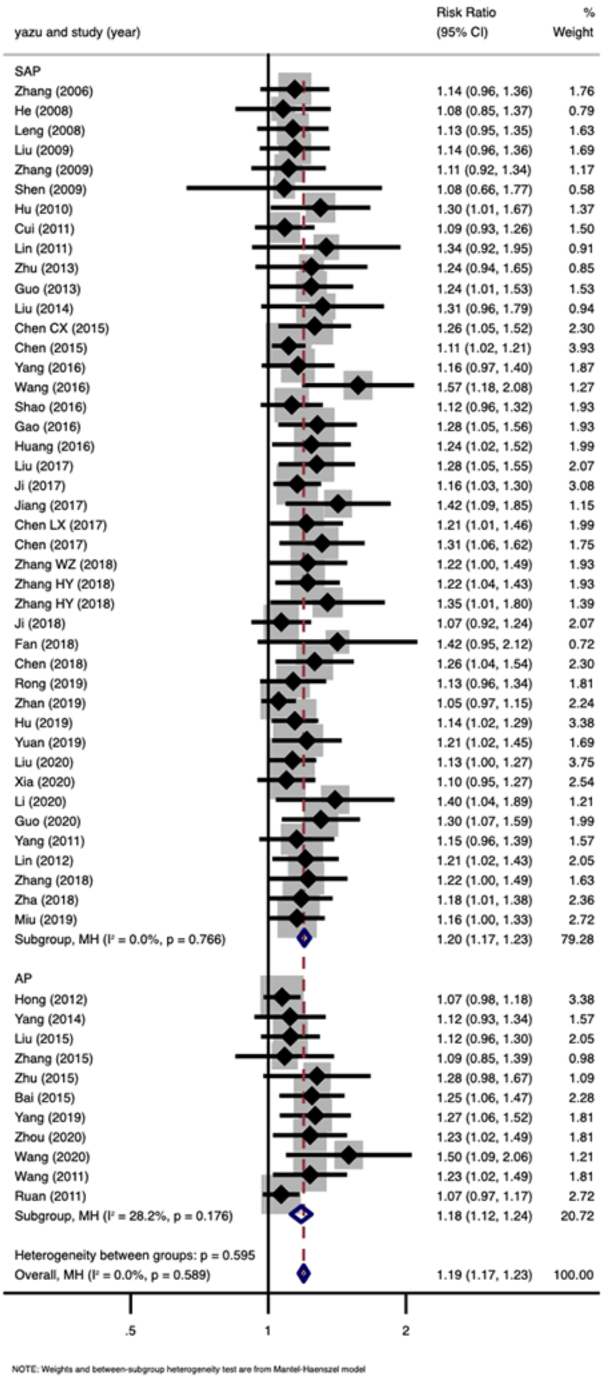

Figure 7 Total effectiveness rate

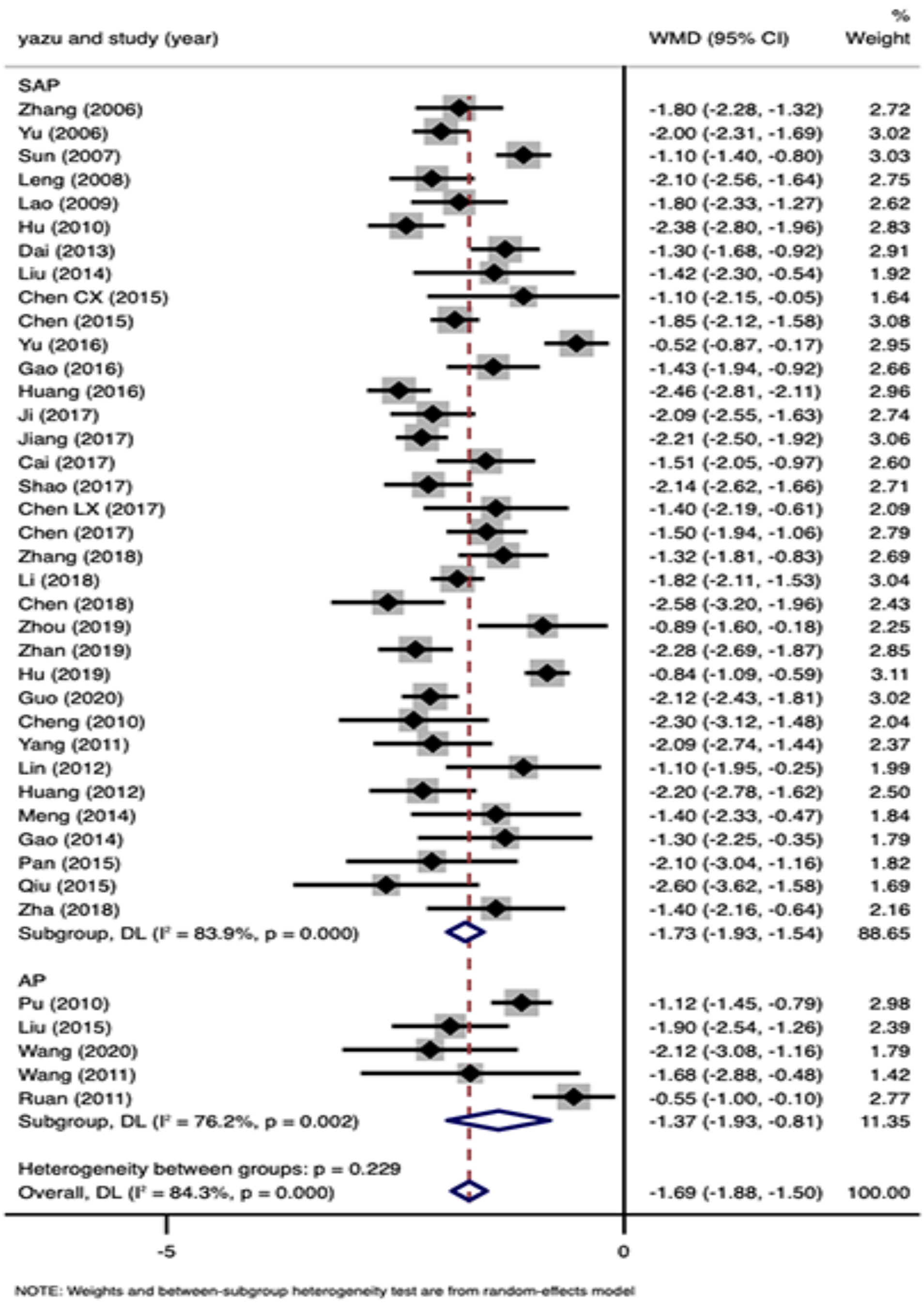

Figure 8 Time until relief of abdominal pain

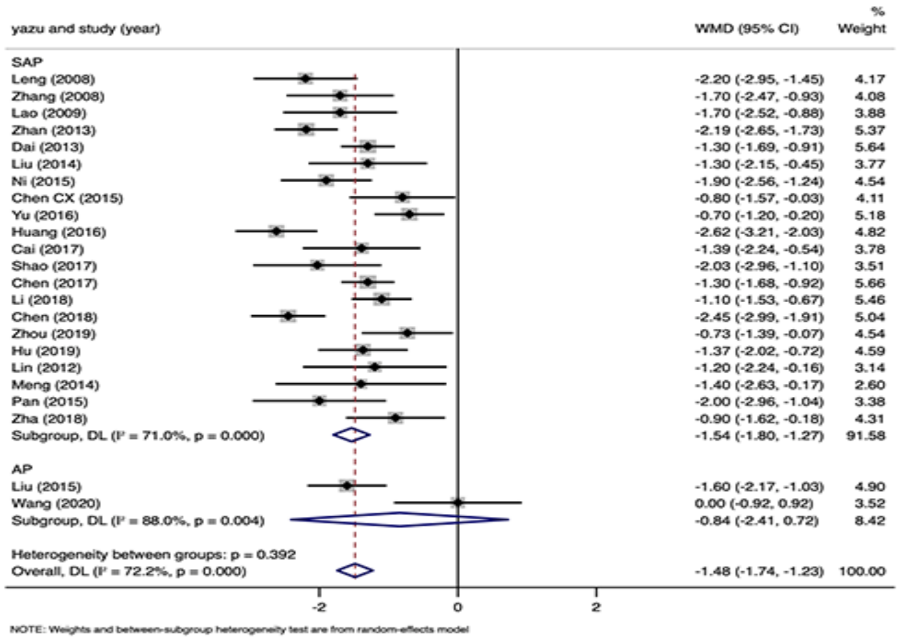

Figure 9 Time until relief of abdominal distension

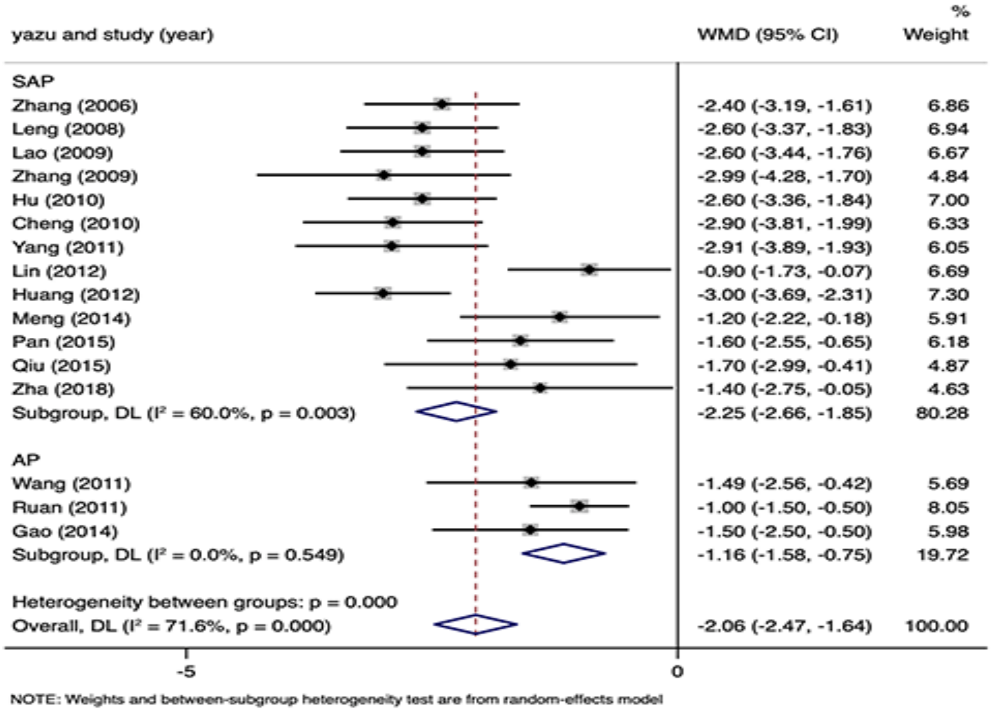

Figure 10 Serum amylase level
