# Supplementary material for: Chinese herbal medicine xuebijing injection for acute pancreatitis: An overview of systematic reviews
Source: Front Pharmacol. 2022 Aug 10;13:883729. doi: 10.3389/fphar.2022.883729 (PMC9399720; doi:10.3389/fphar.2022.883729)
Supplement: Supplementary file 3 [file DataSheet1.PDF]

Supplementary Material

1. Cheng Xia, WANG Yuhong. Clinical observation of xubijing combined with ulinastatin in the treatment of severe acute pancreatitis [J]. Chinese General Practice,2010(24). doi: 10.3969/j.issn.1007-9572.2010.24.005.

2. Hu Gang, Wen Fangtao, Jiang Qimei, et al. Clinical observation of xubijing injection in the treatment of severe acute pancreatitis [J]. Chinese Pharmacy,2010(31).

3. Leng Kai, Huang Zhengtao, Zeng Pengfei. Clinical observation of Xubijing Injection in the treatment of severe acute pancreatitis [J]. China Pharmacy,2008(27).

4. Zejin Pu, Guoping Li, Lingfei Wu. Comparative study on the efficacy of Xubijing combined with magnesium sulfate in the treatment of acute pancreatitis [J]. Jilin Medical Journal,2010(35). doi: 10.3969/j.issn.1004-0412.2010.35.018.

5. Chengkao Sun, Yaochen Bian. Clinical study on the effect of Xubijing on endothelial cell function and plasma endotoxin in patients with severe acute pancreatitis [J]. Modern Distance Education of Chinese Traditional Medicine,2007(10). doi: 10.3969/j.issn.1672-2779.2007.10.023.

6. Yang X F. Effect of Xubijing on plasma endothelin concentration in patients with acute pancreatitis [J]. Medical Information,2010(10). doi: 10.3969/j.issn.1006-1959.2010.10.145.

7. Yu De-gang, Zhuang Bao-xiong, Zhao Peng, et al. Effect of Xubijing Injection on plasma endothelin concentration in severe acute pancreatitis [J]. Chinese Journal of Critical Care Medicine,2006(3). doi: 10.3760/j.issn:1003-0603.2006.03.021.

8. Zhang Wanxiang, Li Zhijun, Wang Jinda. Effect of Xubijing injection on severe acute pancreatitis in 42 cases [J]. Chinese Journal of Emergency Medicine,2006(8). doi: 10.3969/j.issn.1002-1949.2006.08.033.

9. Zha Ling, Gu Yuxing, Zhao Ming. Clinical observation of ulinastatin combined with Xuebijing in the treatment of severe acute pancreatitis [J]. Chongqing Medical Journal,2018(8). doi: 10.3969/j.issn.1671-8348.2018.08.037.

10. Duan Yongqing, Gan Ping, Zhang Jiahua, et al. Effect of Xubijing on inflammatory mediators in patients with acute severe pancreatitis [J]. International Journal of Traditional Chinese Medicine and Chinese Materia Medica,2011(2). doi: 10.3760/cma.j.issn.1673-4246.2011.02.003.

11. Feng Lin, Yan Lin, Jianxiang Hong. Clinical observation of Xubijing injection combined with ulinastatin in the treatment of severe acute pancreatitis [J]. Inner Mongolia Traditional Chinese Medicine,2012(15). doi: 10.3969/j.issn.1006-0979.2012.15.009.

12. Meng Xiaofeng, Wang Hongmin. Effect of Xubijing combined with ulinastatin on serum cytokines in patients with acute severe pancreatitis [J]. Chinese Journal of Advanced Medical Practitioners,2014(5). doi: 10.3760/cma.j.issn.1673-4904.2014.05.019.

13. Miu LI. Effect of ulinastatin and Xuebijing on serum IL-6 and TNF-α levels of patients with severe acute pancreatitis [J]. Contemporary Medical Essays,2019(3).

14. Pan Deng, Sun Yue, FEMALE. Effects of ulinastatin combined with Xuebijing on serum interleukin-8 and interferon γ in patients with severe acute pancreatitis [J]. Chinese Clinical Journal,2015(5). doi: 10.3760/cma.j.issn.1008-6315.2015.05.020.

15. Huaxin Xiao, Kejiang Tang. Effects of Xubijing Injection combined with ulinastatin on serum endotoxin and inflammatory factors in patients with severe acute pancreatitis [J]. Journal of Hainan Medical College,2016(14). doi: 10.13210/j.cnki.jhmu.20160330.003.

16. Zhang Jiexian. Efficacy of Xubijing combined with ulinastatin in the treatment of severe acute pancreatitis and its effect on serum inflammatory factors [J]. China Clinical New Medicine,2018(6). doi: 10.3969/j.issn.1674-3806.2018.06.23.

17. Zhu Qing, Huang Chongfa, Bu Quanhui, et al. Effects of Xubijing on inflammatory factors, vascular endothelial function and hLA-DR expression of monocyte in patients with severe acute pancreatitis [J]. Journal of Zunyi Medical College,2018(4). doi: 10.3969/j.issn.1000-2715.2018.04.014.

18. Zhou-Tao He, Xiang Yang Han. Effects of somatostatin combined with Xuebijing on inflammatory mediators in elderly patients with severe acute pancreatitis [J]. Chinese Journal of Gerontology,2013(18). doi: 10.3969/j.issn.1005-9202.2013.18.067.

19. Sun Changjiang, Fan Hui, Ge Jianbin. Effects of Xubijing on serum levels of tumor necrosis factor -α, interleukin-1 β and interleukin-6 in patients with acute pancreatitis [J]. Journal of Practical Clinical Medicine,2013(9). doi: 10.7619/jcmp.201309029.

20. Zhang Wenyong. Effect of Xubijing Injection combined with somatostatin on cytokines in mild acute pancreatitis [J]. Hebei Medical Journal,2010(7). doi: 10.3969/j.issn.1006-6233.2010.07.020.

21. Dai Xiaogang, Li Shujun, Wang Jianhong, Lu Wei. Effects of Xubijing combined with alanyl glutamine on IL-10, IL-15, IL-18, endotoxin and TNF-α in patients with severe acute pancreatitis [J]. China coal industry medical journal,2013,16(10):1581-1584.

22. Zhihang Lin, Guowei Zhang, Quanquan Zhuang. Effects of Xubijing injection on il-10, IL-15 and IL-18 in patients with severe acute pancreatitis [J]. Chinese journal of clinical pharmacology,2011,27(09):669-671.

23. Wang YI, Gu Xiang, Shao Jialiang, et al. Effects of Xubijing on serum inflammatory factors and oxygen free radicals in patients with acute pancreatitis [J]. Modern Digestion and InterventionalTherapy,2013(5). doi: 10.3961/j.issn.1672-2159.2013.05.006.

24. Shao Jianwei, Zhou Weijun, Sheng Huiqiu, Ni Tongtian, Lu Yiming. Effect of xubijing injection on inflammatory factors in the treatment of severe acute pancreatitis [J]. China journal of new drugs and clinical,2013,32(05):379-382.

25. Chen Jiesheng, Wu Shenfeng, Zhang Yuguang, Li Jia, Zhou Xiaochu. Clinical observation of Dachengqi Decoction combined with Xuebijing Injection in the treatment of early severe acute pancreatitis [J]. Journal of guangzhou university of traditional Chinese medicine,2013,30(03):305-308.

26. Qi Chen, Hongxiang Yin. Effect of anisodamine combined with Xuebijing in the treatment of severe pancreatitis [J]. World Chinese Journal of Digestion,2015(21).

27. Ma Dengchao, Li Yong. Effects of Xubijing Injection on serum inflammatory cytokines and hemorheology of severe acute pancreatitis [J]. Chinese Medical Emergency,2015(2). doi: 10.3969/j.issn.1004-745X.2015.02.067.

28. Qiu Ying, Zhang Jun, Bai Maqiao, et al. Application of ulinastatin combined with Xuebijing in acute severe pancreatitis [J]. Chinese and Foreign Medical Research,2015(21). doi: 10.14033/j.cnki.cfmr.2015.21.012.

29. Wang Zhongheng, Xiao Shunguo, Li Dongbai. Clinical Observation of ulinastatin combined with Xuebijing in the treatment of acute pancreatitis [J]. Clinical Practice of Integrated Traditional Chinese and Western Medicine,2011(2). doi: 10.3969/j.issn.1671-4040.2011.02.012.

30. Zeng Jie, Chen Ningbo. Effect of ulinastatin combined with Xuebijing on immune factors in patients with severe acute pancreatitis [J]. Clinical Journal of Practical Hospital,2013(5). doi: 10.3969/j.issn.1672-6170.2013.05.040.

31. Gao Rongkai. Efficacy analysis of ulinastatin combined with Xuebijing in the treatment of acute pancreatitis [J]. China Practical Medicine,2014(23).

32. Zhiwen Huang, Huiwen Yao. Efficacy of ulinastatin combined with Xuebijing in the treatment of acute pancreatitis [J]. Chinese Journal of Physicians,2012(Z1).

33. Ruan Ming-wen. Clinical study of ulinastatin combined with Xuebijing injection in the treatment of acute pancreatitis [J]. J Clinical Rational Drug Use,2011(32). doi: 10.3969/j.issn.1674-3296.2011.32.037.

34. Yang Yongjun. Clinical analysis of xubijing combined with ulinastatin in the treatment of severe acute pancreatitis [J]. Chinese Journal of Misdiagnosis,2011(24).

35. Liu Dezhi, Wang Jinlei. Therapeutic effect of Xubijing injection on severe acute pancreatitis [J]. Journal of Xinxiang Medical College,2009(5).

36. Zhu Kedong. Clinical observation of xubijing injection in treatment of severe acute pancreatitis [J]. Chin J general practice,2013,11(07):1054+1074.

37. Zhang Xiaofeng, Xu Huaping, Zhao Guohai. Effects of Xuebijing Injection on serum TNF-α and IL-10 in patients with severe acute pancreatitis [J]. Zhejiang Clinical Medicine,2009(2). doi: 10.3969/j.issn.1008-7664.2009.02.003.

38. Wang Xiaoqiu, Yu Weizhou, Ji Gan. Analysis of the therapeutic effect of Xubijing on severe acute pancreatitis [J]. Clinical Meta-analysis,2009(17).

39. Haitao Shen, Feng Guo, Min Zhao. Chinese Journal of Integrated Traditional and Western Medicine First Aid,2009(3). doi: 10.3969/j.issn.1008-9691.2009.03.021.

40. Qingju He, Qingwen Li. Effect of Xubijing injection on severe acute pancreatitis [J]. Chinese Journal of Modern Integrated Traditional and Western Medicine,2008(34). doi:

10.3969/j.issn.1008-8849.2008.34.012.

41. Cui Junke, Cao Songyan, Liu Hongwei. Clinical analysis of xubijing injection in treatment of 56 cases of severe acute pancreatitis [J]. Chinese and Foreign Health Digest,2011(22). doi: 10.3969/j.issn.1672-5085.2011.22.074.

42. Cheng Xia, Zhou Rongbin. Clinical observation of Xuebijing injection in the treatment of acute severe pancreatitis [C]. Chinese Association of Integrated Traditional and Western Medicine Emergency Medicine Professional Committee.2005 National Academic Conference of critical emergency Medicine academic proceedings. Emergency Medicine Professional Committee of Chinese Association of Integrated Traditional and Western Medicine: Chinese Association of Integrated Traditional and Western Medicine,2005:192-193.

43. Zhiyong Lao, Qi Zeng, Bai Xing. Study on the correlation of Xubijing in the treatment of severe acute pancreatitis [J]. Shandong Medicine,2009(8). doi: 10.3969/j.issn.1002-266X.2009.08.039.

44. Hong Liming, Zhan Yixing, Pan Ximing. Clinical observation of Xubijing Injection in the treatment of acute pancreatitis and its effect on serum hypersensitive C-reactive protein [J]. Chinese Medical Emergency,2012(6). doi: 10.3969/j.issn.1004-745X.2012.06.066.

45. Jinsong Ren, Yong Zheng, Liangke Li. The relationship between TNF-α and IL-6 in acute necrotizing pancreatitis and the role of Xuebijing [J]. Medical Informatics,2013(1). doi: 10.3969/j.issn.1006-1959.2013.01.115.

46. Jinliang Yang, Rongwen Zhang, Haimo Chen. Treatment of 31 cases of acute pancreatitis with Xuebijing Injection [J]. Fujian Traditional Chinese Medicine,2014(4).

47. Liu Wanli. Effects of Xubijing Injection on plasma endothelin and ammonia monoxide levels in patients with severe pancreatitis [J]. Journal of Modern Integrated Chinese and Western Medicine,2014(7). doi: 10.3969/j.issn.1008-8849.2014.07.017.

48. Deng JIANbo, Shen Li, Shi Wanping, et al. Clinical observation of Acute renal injury induced by Acute severe pancreatitis with Xuebijing Injection [J]. Chinese Medical Emergency,2015(6). doi: 10.3969/j.issn.1004-745X.2015.06.051.

49. Chen Hong, Wang Suli, Zhu Kunpeng. Effect of Xubijing Injection in the adjuvant treatment of acute severe pancreatitis [J]. China Pharmacy,2015(32). doi: 10.6039/j.issn.1001-0408.2015.32.30.

50. Bai Yanhua, Liang Yuping. Clinical analysis of 50 cases of acute pancreatitis treated by Xuebijing [J]. Chinese Medical Journal,2015(10). doi: 10.3969/j.issn.1674-0742.2015.10.055.

51. Liu Shang, Li Zhiyong. Clinical observation of Xubijing Injection combined with Western medicine in the treatment of acute pancreatitis [J]. Chinese Journal of Traditional Chinese Medicine Emergency,2015(4). doi: 10.3969/j.issn.1004-745X.2015.04.062.

52. Chen Caixia, Xie Ping. Clinical study of Xubijing Injection combined with Conventional

Treatment of Western Medicine in the treatment of severe acute pancreatitis [J]. International Journal of Chinese Medicine and Materia Medica,2015(5). doi: 10.3760/cma.j.issn.1673-4246.2015.05.005.

53. Wang Xue, Wang Xiankun, Cen Rongfei, et al. Effect of Xubijing Injection on the efficacy and cellular immune function of severe acute pancreatitis [J]. Chinese Journal of New Drugs and Clinical Medicine,2015(6). doi: 10.14109/j.cnki.xyylc.2015.06.016.

54. Zhang Li. Study and analysis of xuebijing in the treatment of acute pancreatitis [J]. Medical Information,2015(23). doi: 10.3969/j.issn.1006-1959.2015.23.347.

55. Ni Haoliang. Treatment of acute hemorrhagic necrotizing pancreatitis with Xubijing combined with testameptide in 30 cases [J]. Medical Review,2015(16). doi: 10.3969/j.issn.1006-2084.2015.16.067.

56. Zhang MEIhua, Huang Jianwei, Wen Ling, et al. Effect of Xubijing combined with blood purification on acute severe pancreatitis [J]. Journal of Qiqihar Medical College,2015(10).

57. Zhu Li. Clinical effect of somatostatin and pantoprazole combined with Xuebijing in the treatment of acute pancreatitis [J]. World Clinical Medicine,2015(10).

58. Shao Zhongyi, Su Saisai, Borisa. Clinical efficacy of Xubijing in the treatment of severe pancreatitis and its effect on coagulation function [J]. World Chinese Journal of Digestion,2016(25). doi: 10.11569/wcjd.v24.i25.3712.

59. Yu Y. The effect of Xubijing on clinical efficacy, oxidative stress, CRP, IL-6 and TNF-α in acute pancreatitis [J]. Journal of Hainan Medical College,2016(14). doi: 10.13210/j.cnki.jhmu.20160414.013.

60. Zhang Guangyou. Clinical effect of Xubijing combined with blood purification on acute severe pancreatitis [J]. Journal of Qiqihar Medical College,2016(16).

61. Peng Gao, Zhansheng Hu. Clinical observation of xubijing combined with somatostatin in the treatment of severe acute pancreatitis [J]. Proprietary Chinese medicine, 2016, (3). doi: 10.3969/j.issn.1001-1528.2016.03.055.

62. Wang Wenju, Su Weixiang, Lin Dan. Efficacy of Xubijing combined with somatostatin for injection in the treatment of severe acute pancreatitis with Liver stagnation and fire [J]. Chinese Materia Medica Pharmacology and Clinical,2016(4).

63. Huang P, Huang Z, Qin W B, et al. Clinical observation of Xubijing Injection in the adjuvant treatment of severe acute pancreatitis [J]. China Pharmacy,2016(32). doi: 10.6039/j.issn.1001-0408.2016.32.38.

64. Yang Kun, Zhao Jingcheng. Protective effect of Xubijing on acute kidney injury caused by severe acute pancreatitis [J]. Chinese Journal of Integrated Traditional and Western Medicine Digestion,2016(10). doi: 10.3969/j.issn.1671-038X.2016.10.10.

65. Liu Xiangdong. Effects of Chinese medicine injection on hS-CRP, IL-6 and TNF-α levels of pancreatitis and its efficacy evaluation [J]. World Chinese Digestive Journal,2017(10). doi: 10.11569/wcjd.v25.i10.929.

66. Chen Keping, Zheng Yong. Analysis of endotoxin clearance and protective mechanism of renal injury in patients with severe pancreatitis by Xuebijing [J]. Shanxi Medical Journal,2017(8). doi: 10.3969/j.issn.0253-9926.2017.08.035.

67. Chen Lixia, Zhang Yanping. Clinical observation of xubijing combined with somatostatin in the treatment of severe acute pancreatitis [J]. Modern Diagnosis and Therapy,2017(14). doi: 10.3969/j.issn.1001-8174.2017.14.029.

68. Shao Zhongyi, Su Saisai, Paulisa. Clinical observation of Xubijing injection in adjuvant treatment of severe acute pancreatitis [J]. New Traditional Chinese Medicine,2017(2). doi: 10.13457/j.cnki.jncm.2017.02.017.

69. Ji Huaqing, Yang Ping. Clinical effect of Xubijing Injection combined with ulinastatin on serum inflammatory factors in patients with severe acute pancreatitis [J]. Shaanxi Traditional Chinese Medicine,2017(7). doi: 10.3969/j.issn.1000-7369.2017.07.048.

70. CAI Chao-shen. Clinical observation of Xubijing injection combined with somatostatin in the treatment of severe pancreatitis [J]. Chinese Contemporary Medicine,2017(11). doi: 10.3969/j.issn.1674-4721.2017.11.012.

71. Jiang Zhengcai, Dong Jianda, Chen Tong. Clinical observation of somatostatin and pantoprazole sodium as base drug combined with other drugs in the treatment of acute severe pancreatitis [C]. 2017.

72. Fan Y. Effect of Xubijing combined with Esomeprazole in the treatment of severe acute pancreatitis [J]. Journal of Clinical Medicine Literature,2018(39). doi: 10.3877/j.issn.2095-8242.2018.39.016.

73. Chen Jia-lian, CAI Yan-xing, Chen Ke-shu, et al. Effect of Xubijing Injection on oxidative stress and lung injury in severe acute pancreatitis [J]. Progress of Modern General Surgery in China,2018(12). doi: 10.3969/j.issn.1009-9905.2018.012.020.

74. Li Yi, Zhou Ping. Effect of Xubijing combined with somatostatin on TG, inflammatory parameters and prognosis of severe high-fat pancreatitis [J]. Journal of North Sichuan Medical College,2018(5). doi: 10.3969/j.issn.1005-3697.2018.05.014.

75. Ji Xinzun, Hu Qingfu, Zhang Youshuai. Application of Xubijing Injection in severe acute pancreatitis complicated with sepsis [J]. Hainan Medical Journal,2018(23). doi: 10.3969/j.issn.1003-6350.2018.23.021.

76. Zhang Wenzhao, Luo Dongxu. Effects of Xubijing Injection on serum HS-CRP, IL-1 and IL-6 levels in patients with acute severe acute pancreatitis [J]. Strait Pharmacy,2018(12). doi: 10.3969/j.issn.1006-3765.2018.12.071.

77. Chen K. Clinical observation on the efficacy of Xubijing injection combined with ulinastatin and serum inflammatory factors in severe acute pancreatitis [J]. Journal of Aerospace Medicine,2018(1). doi: 10.3969/j.issn.2095-1434.2018.01.035.

78. Zhang HONGying, Zhang Ningning, Niu Dan, et al. Clinical observation of Xubijing combined with Esomeprazole in the treatment of severe acute pancreatitis [J]. Chinese

Journal of Modern Integrated Traditional and Western Medicine,2018(1). doi: 10.3969/j.issn.1008-8849.2018.01.

79. Zhang Hongying, Wang Chan, Zhang Yi, et al. Effect of silamin combined with Xuebijing in the treatment of severe acute pancreatitis and its effect on serum cytokine levels [J]. Western Medicine,2018(12). doi: 10.3969/j.issn.1672-3511.2018.12.014.
80. Yang YU. Clinical observation of acute pancreatitis in gastroenterology [J]. Doctors, 2019 (8).
81. Chang XIAO, Wang Ling, Chen Junteng, et al. Treatment of severe pancreatitis with acute respiratory distress syndrome by Xubijing Injection [J]. Chinese Medicine Bulletin,2019(3).
82. Yunming Hu, Yunfeng Shan, Peipei Huang. Efficacy of ulinastatin combined with Xuebijing in treatment of severe acute pancreatitis [J]. Chinese Journal of Integrated Traditional and Western Medicine Surgery,2019(5). doi: 10.3969/j.issn.1007-6948.2019.05.004.
83. Rong Shi-yun,QINZHOU,GUANGXI [5]3[5]0[0]0). Efficacy of continuous renal replacement therapy combined with Xuebijing in the treatment of severe acute pancreatitis [J]. World Latest Medical Information Abstracts (Electronic edition),2019(19).
84. Yuan Benquan. Clinical value analysis of Xubijing injection in the treatment of severe acute pancreatitis [J]. Chinese And Foreign Medical Research,2019(5). doi: 10.14033/j.cnki.cfmr.2019.05.014.
85. Zhan Yi, Xu Fen, Fang Zhi. Efficacy of Xubijing Injection combined with octreotide and ulinastatin in the treatment of acute severe pancreatitis and its effect on immune function [J]. Chinese Journal of Traditional Chinese Medicine,2019(5). doi: 10.13193/j.issn.1673-7717.2019.05.060.
86. Zhou B. Clinical effect of Xubijing injection combined with continuous renal replacement therapy on severe acute pancreatitis [J]. Henan Medical Research,2019(22). doi: 10.3969/j.issn.1004-437X.2019.22.056.
87. Wang Zhongxin. Effect of Esomeprazole combined with Xubijing on gastrointestinal function recovery in patients with acute pancreatitis [J]. Henan Medical Research,2020(12). doi: 10.3969/j.issn.1004-437X.2020.12.056.
88. Wu Yingjie. Clinical efficacy of acute pancreatitis in gastroenterology [J]. Health Care Guide,2021(7).
89. Li Guosheng, Lv Yanheng. Effects of Xuebijing injection on hemorheology and TNF-α, IL-6 levels in patients with severe acute pancreatitis [J]. Bright Traditional Chinese Medicine,2020(8). doi: 10.3969/j.issn.1003-8914.2020.08.017.
90. Xia Bing-jie, Qiang Zhan-Rong, Li Gui-xian, et al. Effect of Xuebijing injection on the expression of NLRP3 inflammasome in peripheral blood of elderly patients with severe acute pancreatitis [J]. Chinese Journal of Gerontology,2020(1). doi: 10.3969/j.issn.1005-9202.2020.01.025.

91. Liu Ke. Observation of the clinical efficacy of Xubijing injection combined with octreotide and ulinastatin in the treatment of acute severe pancreatitis [J]. Health Vision,2020(7).

92. Guo Shaoyong, Liu Zhaoxiu, Bao Baijun, et al. Objective To explore the clinical efficacy of xubijing combined with somatostatin in the treatment of severe acute pancreatitis [J]. China Practical Medicine,2020(32). doi: 10.14163/j.cnki.11-5547/r.2020.32.053.
